# Supplementary figures and images for: Uncovering the electrical synapse proteome in retinal neurons via in vivo proximity labeling
Source: eLife. 2026 May 20;14:RP105935. doi: 10.7554/eLife.105935 (PMC13189625; doi:10.7554/eLife.105935)

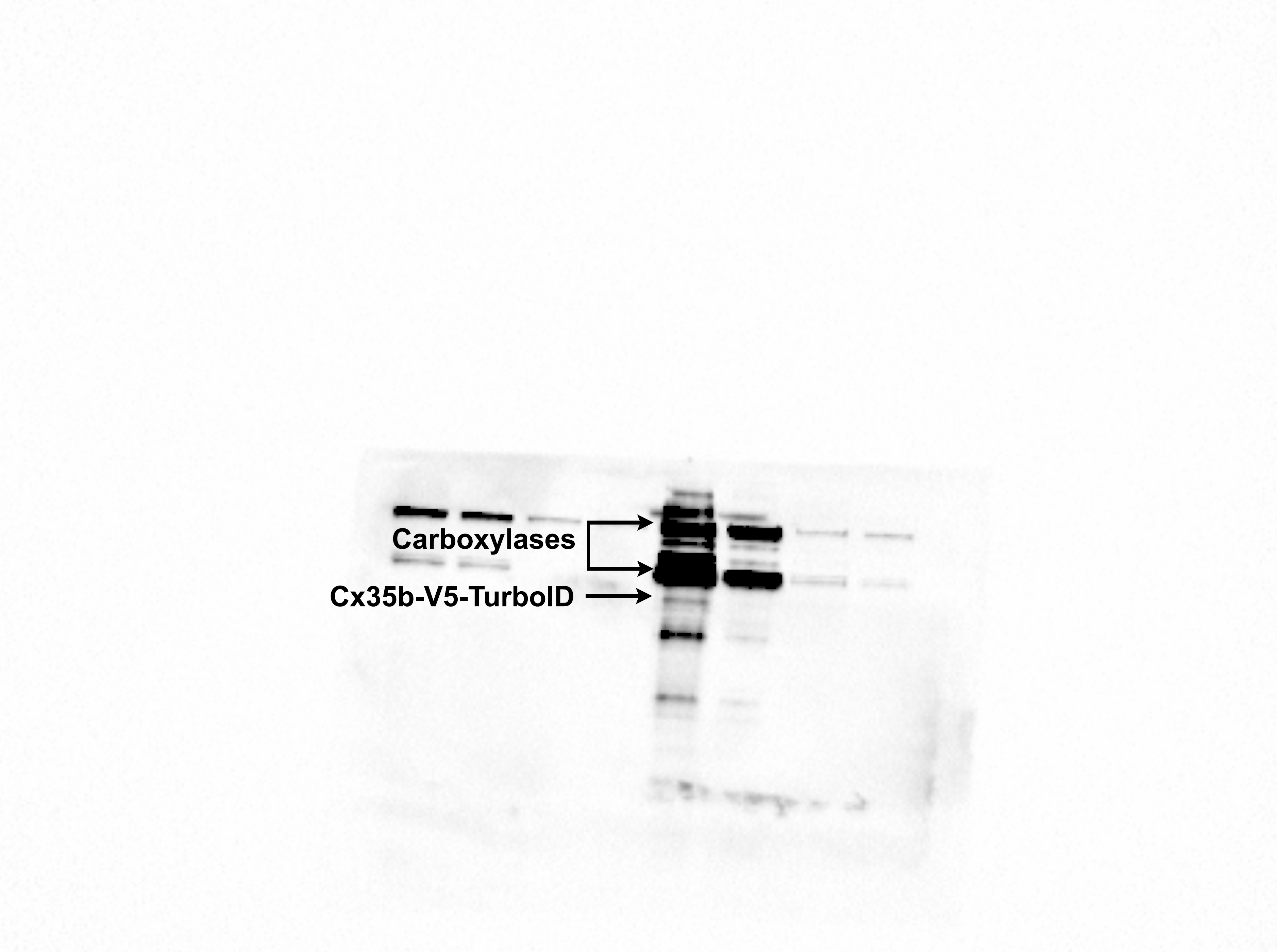

Supplement: Figure 1—source data 1. — Relevant bands were labeled with arrows. [file elife-105935-fig1-data1.zip › Labelled blots/Figure 1C_Streptavidin_labelled.tif]

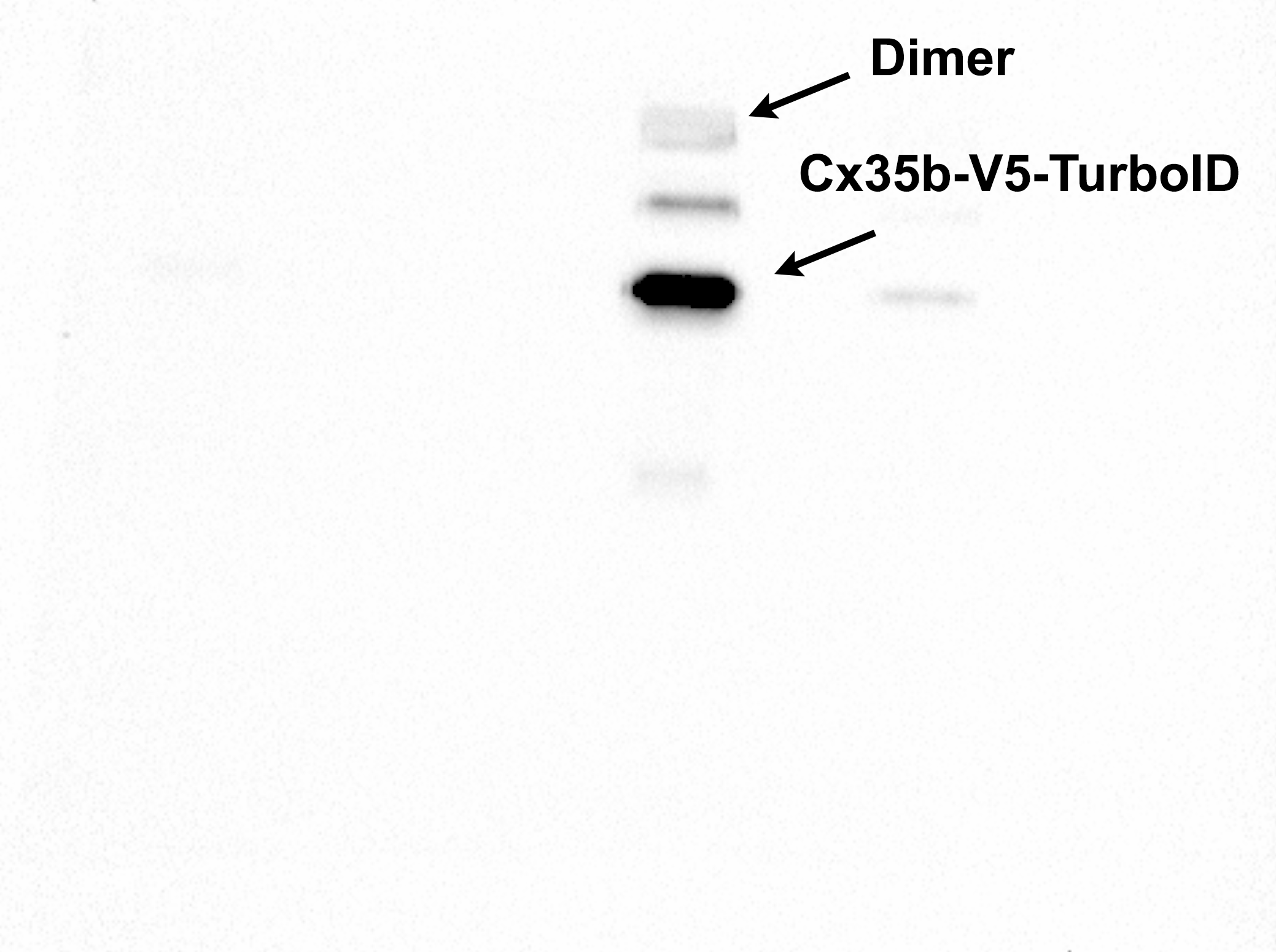

Supplement: Figure 1—source data 1. — Relevant bands were labeled with arrows. [file elife-105935-fig1-data1.zip › Labelled blots/Figure1C_V5_labelled.tif]

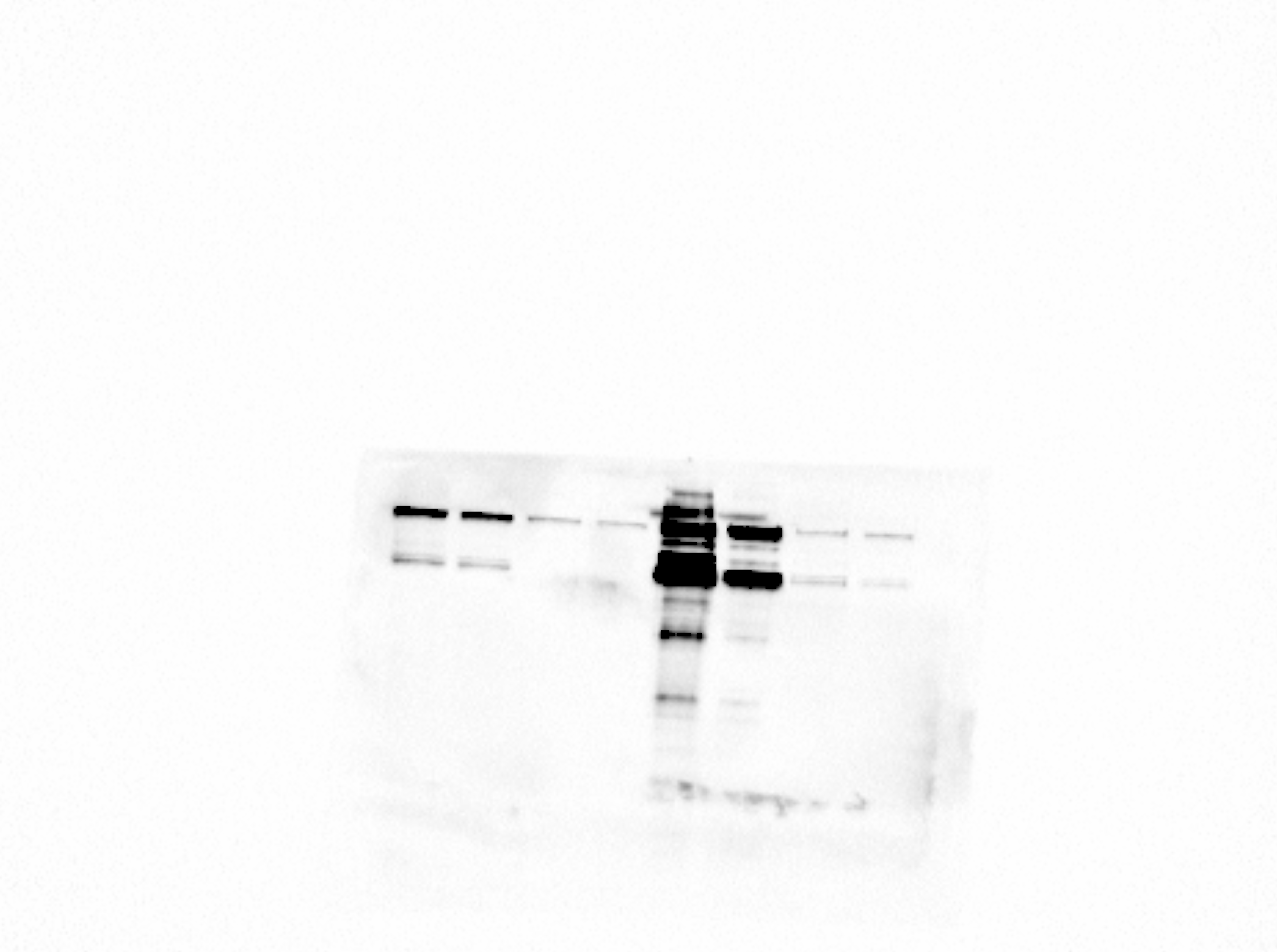

Supplement: Figure 1—source data 2. [file elife-105935-fig1-data2.zip › Unlabelled blots/Figure 1C_ Streptavidin.tif]

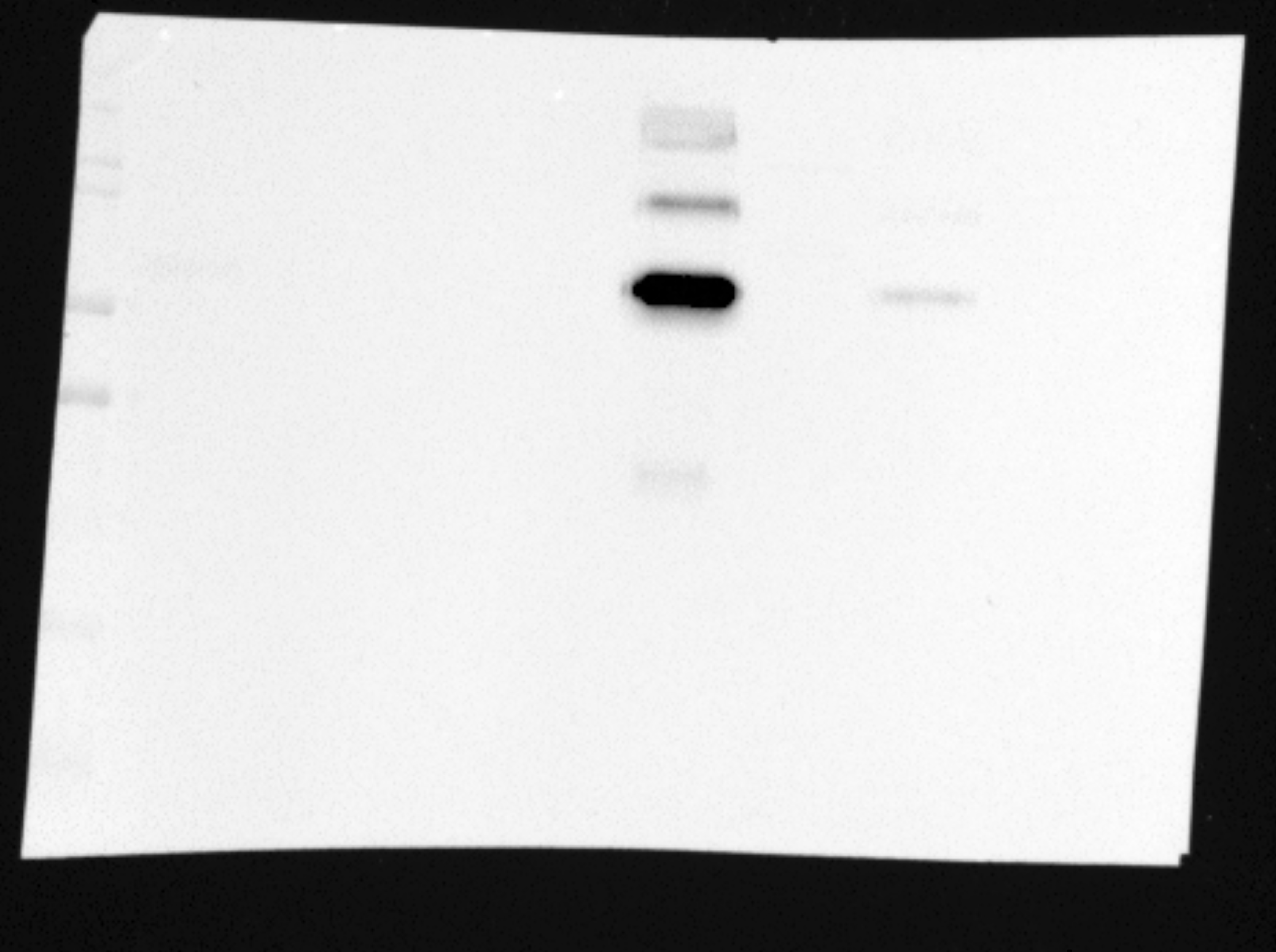

Supplement: Figure 1—source data 2. [file elife-105935-fig1-data2.zip › Unlabelled blots/Figure 1C_V5 with marker.tif]

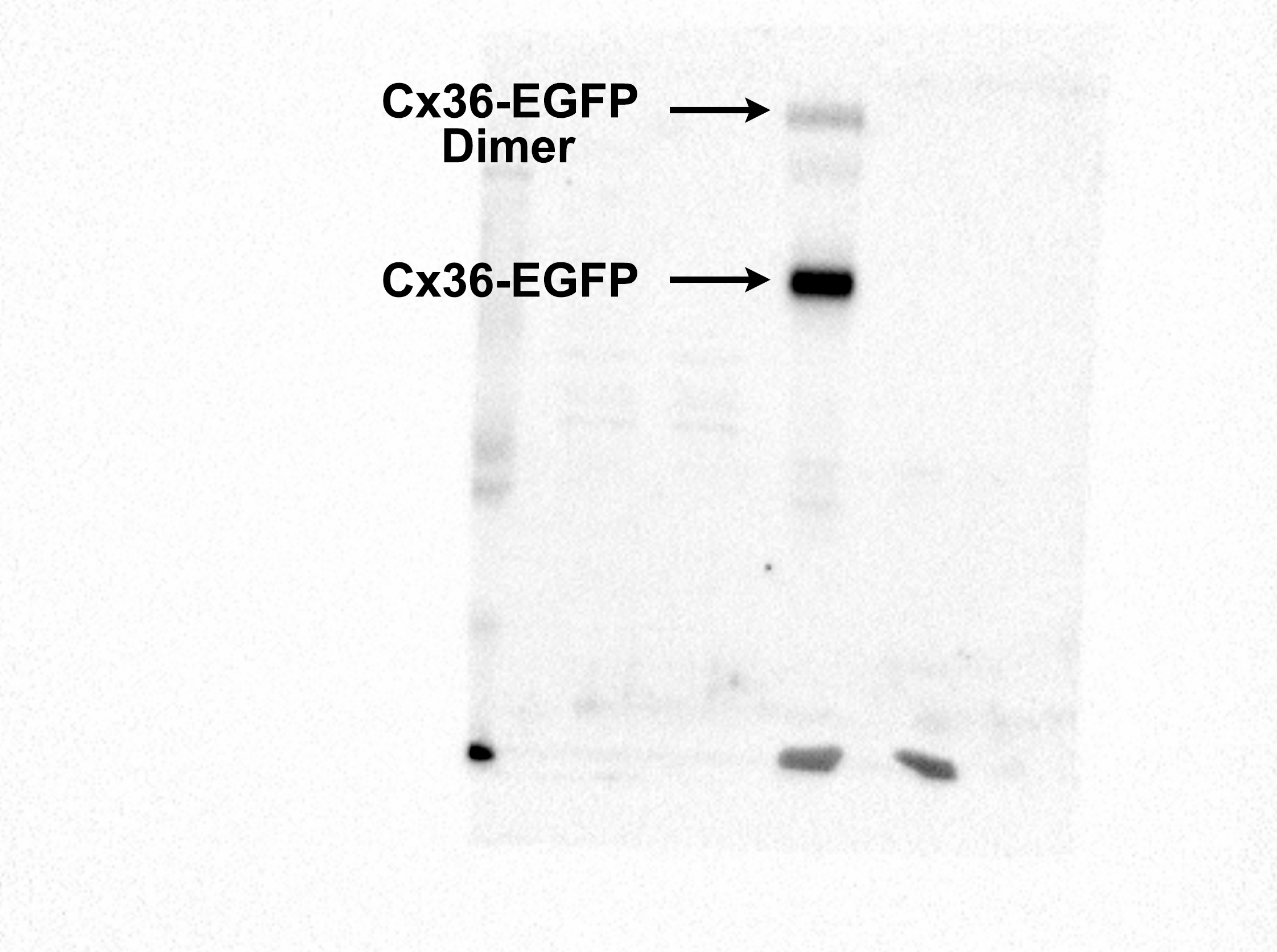

Supplement: Figure 2—source data 1. — Relevant bands were labeled with arrows. [file elife-105935-fig2-data1.zip › Labelled blots/Figure 2C_GFP_labelled.tif]

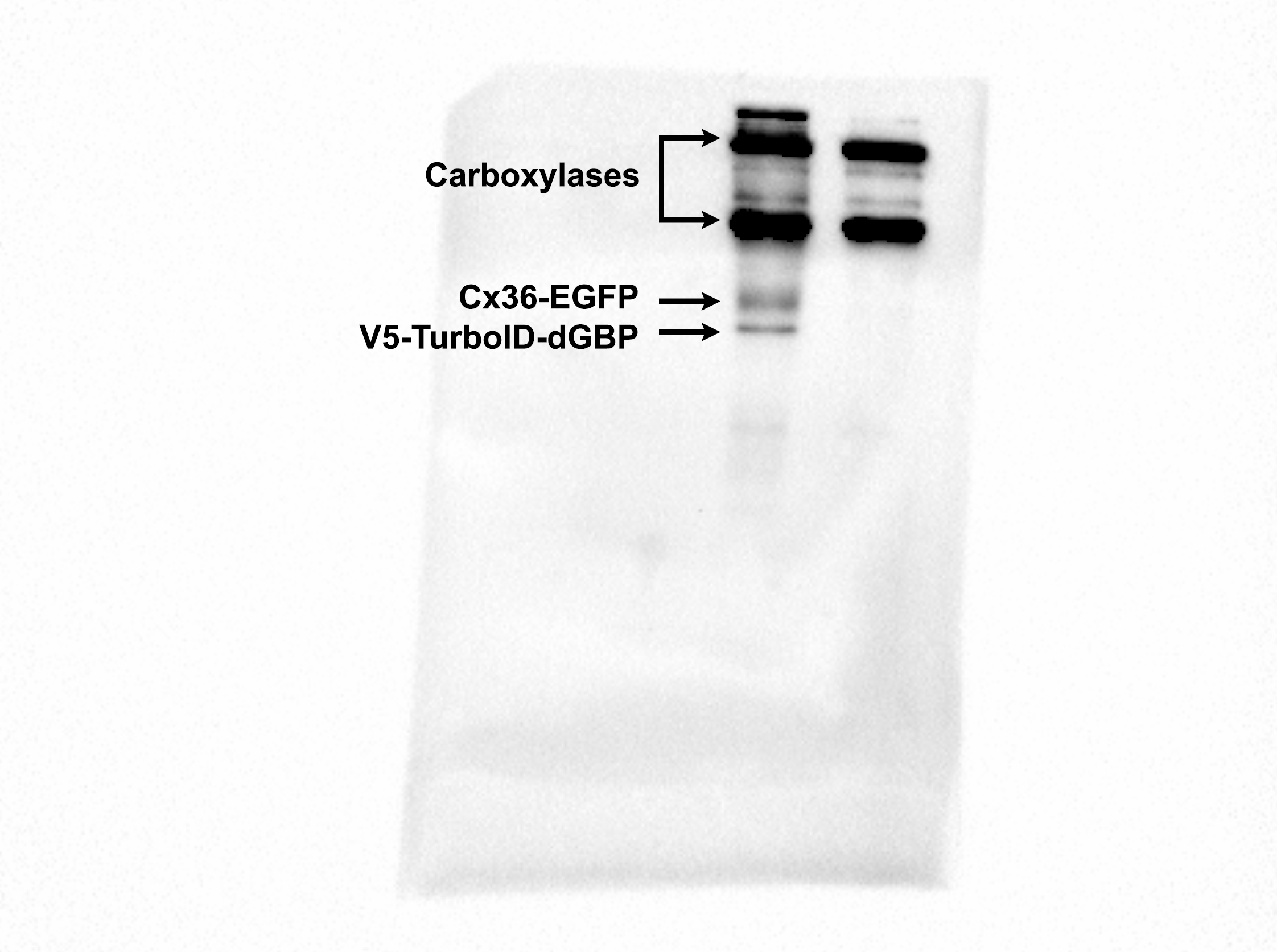

Supplement: Figure 2—source data 1. — Relevant bands were labeled with arrows. [file elife-105935-fig2-data1.zip › Labelled blots/Figure 2C_Streptavidin_labelled.tif]

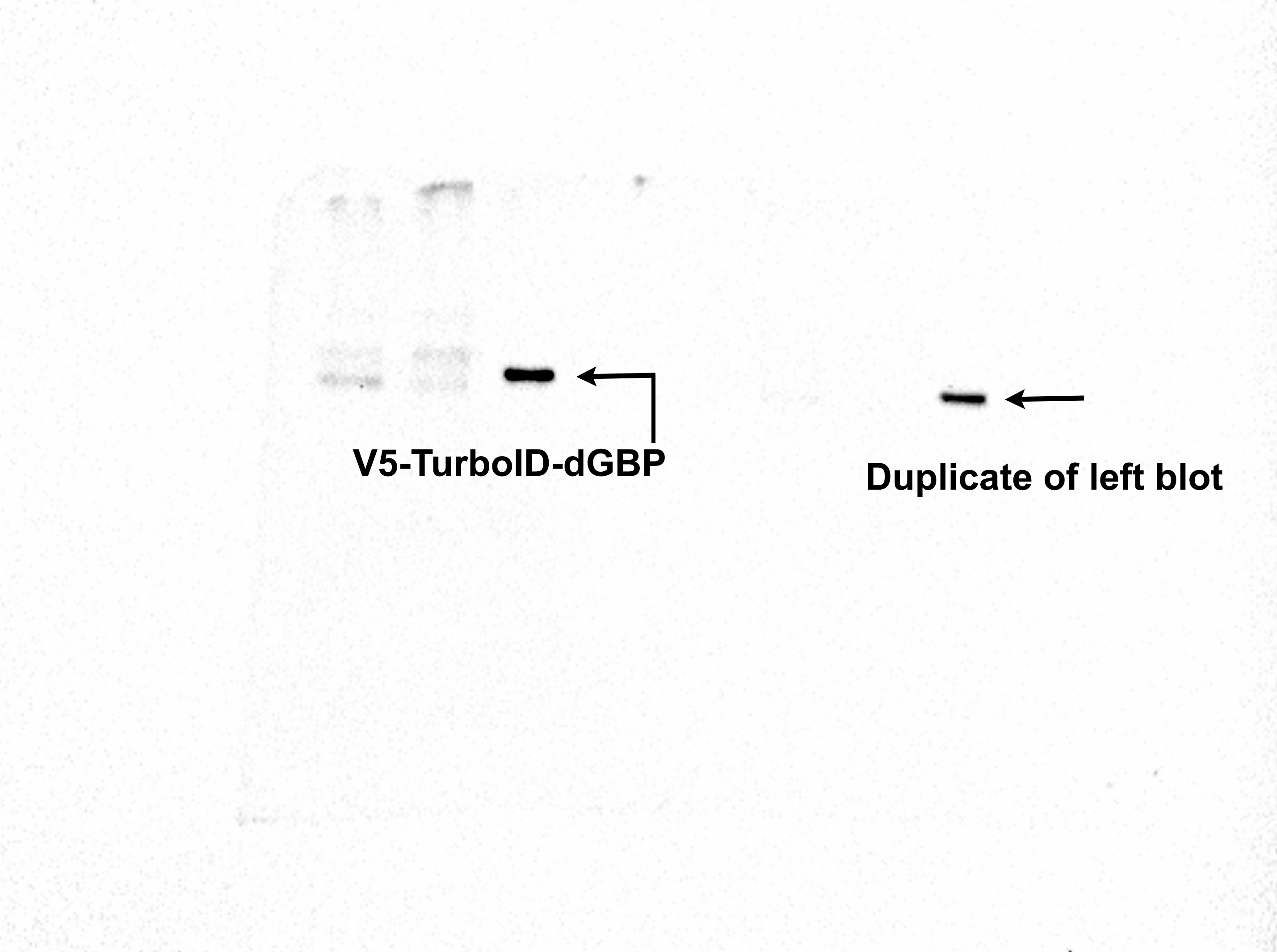

Supplement: Figure 2—source data 1. — Relevant bands were labeled with arrows. [file elife-105935-fig2-data1.zip › Labelled blots/Figure 2C_V5 labelled.tif]

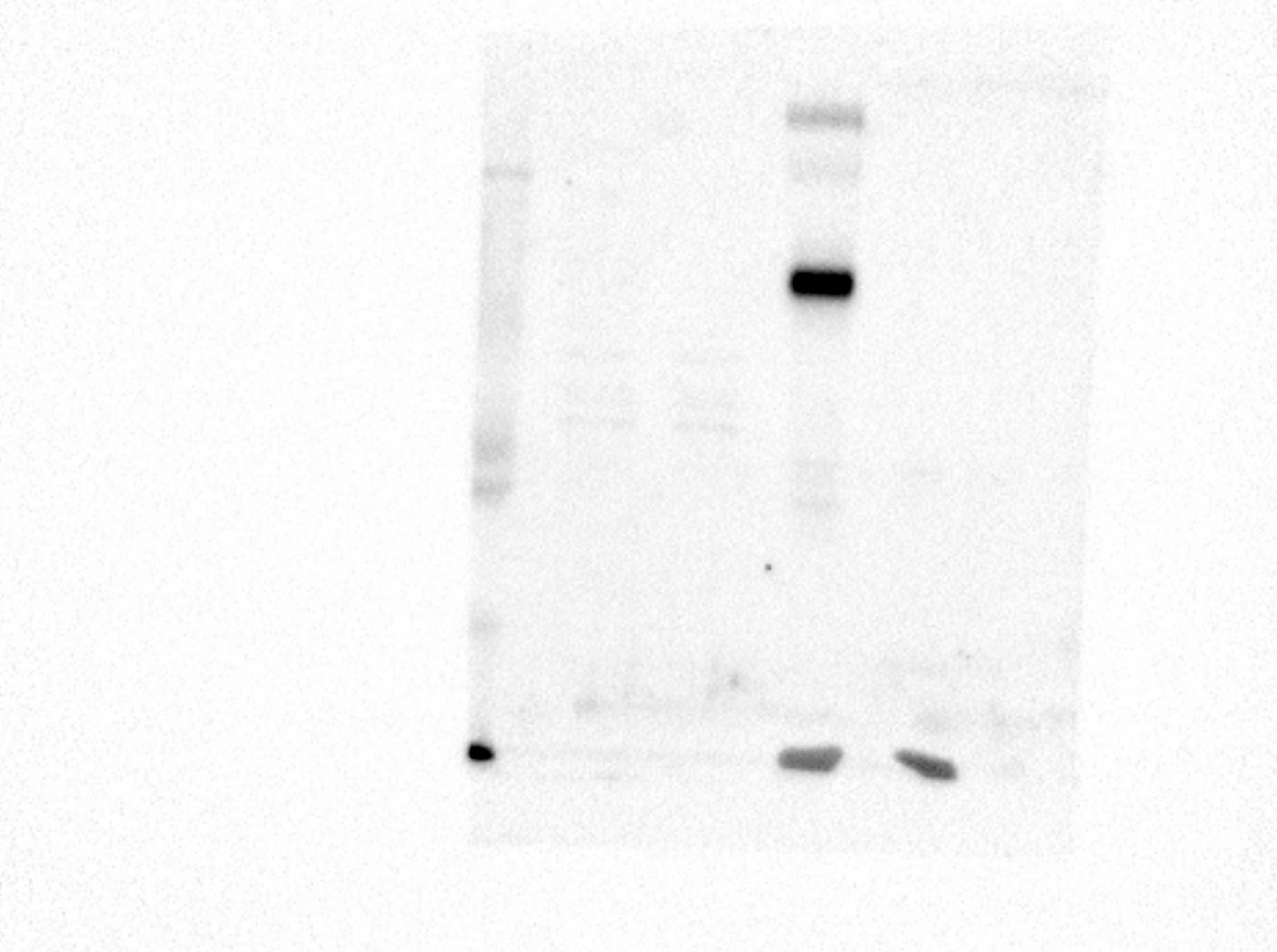

Supplement: Figure 2—source data 2. [file elife-105935-fig2-data2.zip › Unlabelled blots/Figure 2C_GFP.tif]

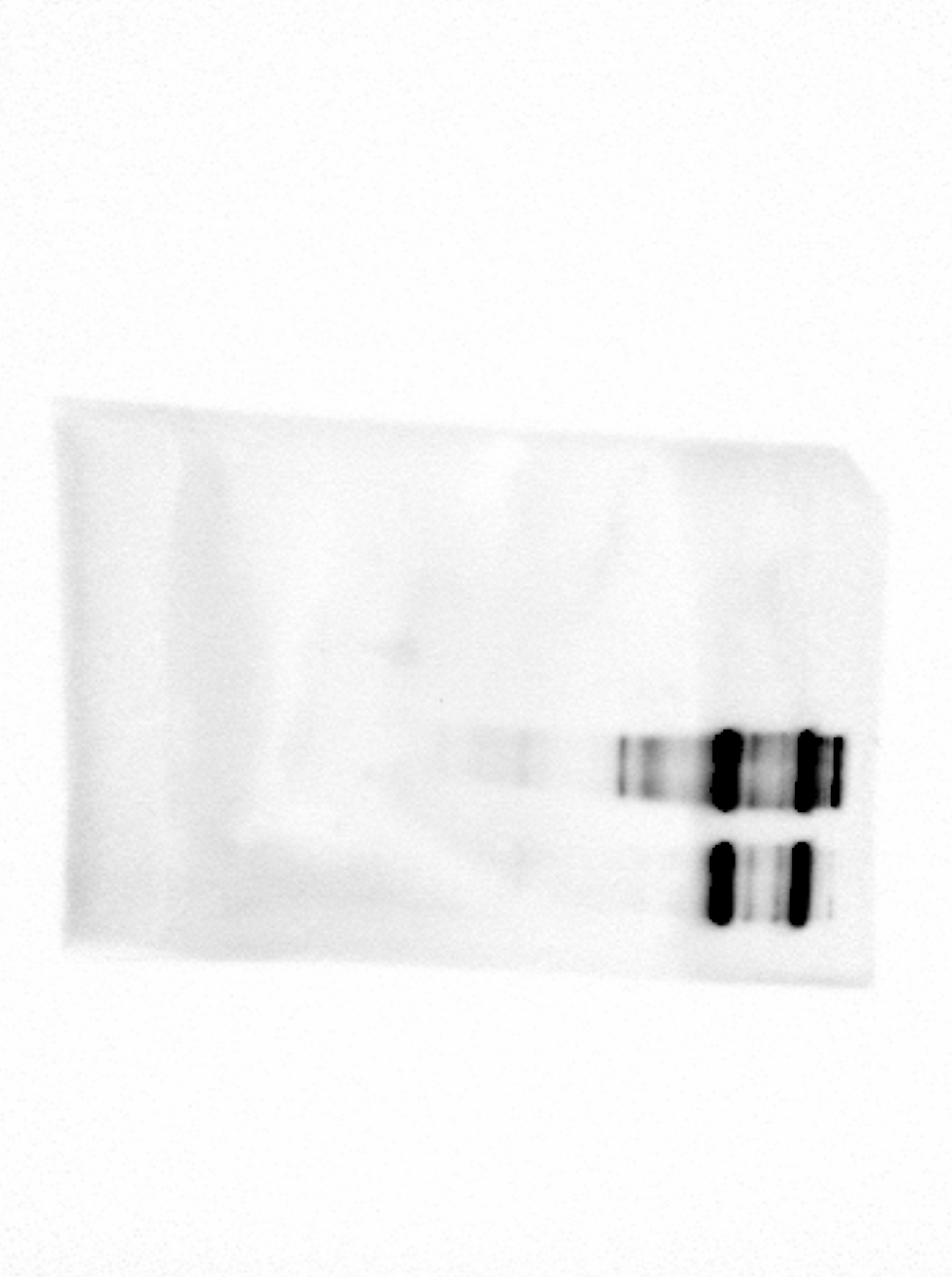

Supplement: Figure 2—source data 2. [file elife-105935-fig2-data2.zip › Unlabelled blots/Figure 2C_Streptavidin.tif]

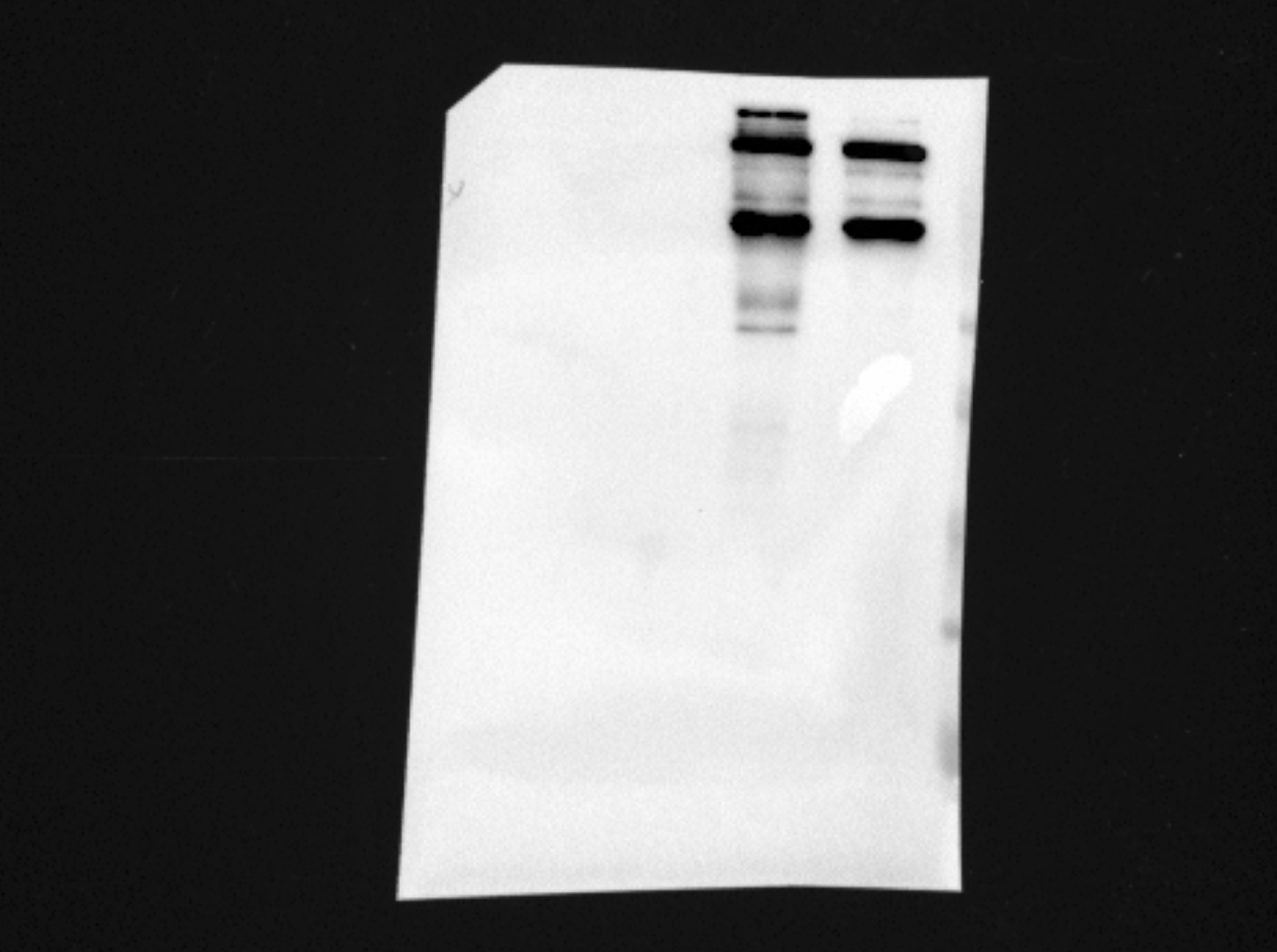

Supplement: Figure 2—source data 2. [file elife-105935-fig2-data2.zip › Unlabelled blots/Figure 2C_Streptavidin_Marker merg.tif]

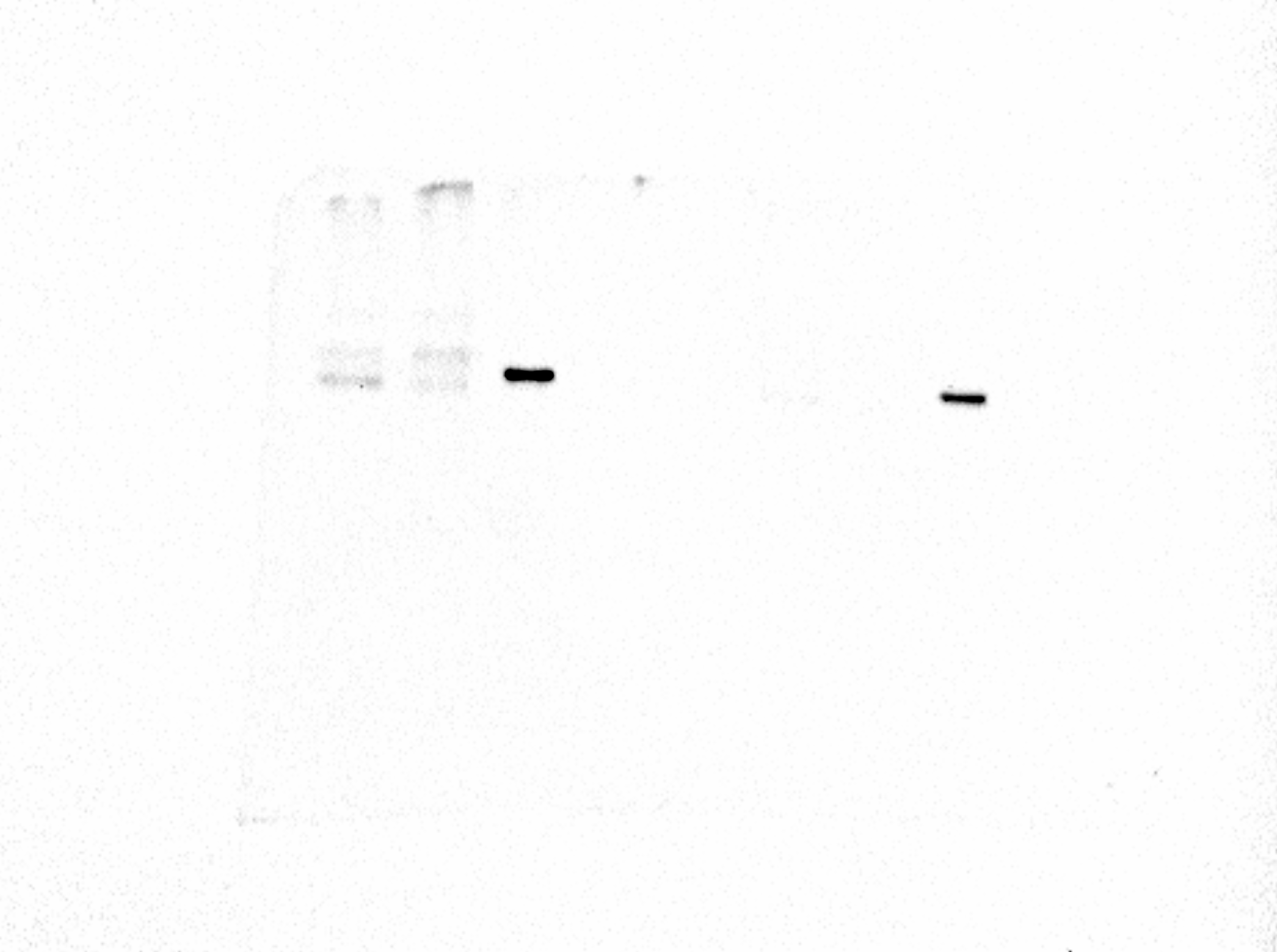

Supplement: Figure 2—source data 2. [file elife-105935-fig2-data2.zip › Unlabelled blots/Figure 2C_V5.tif]

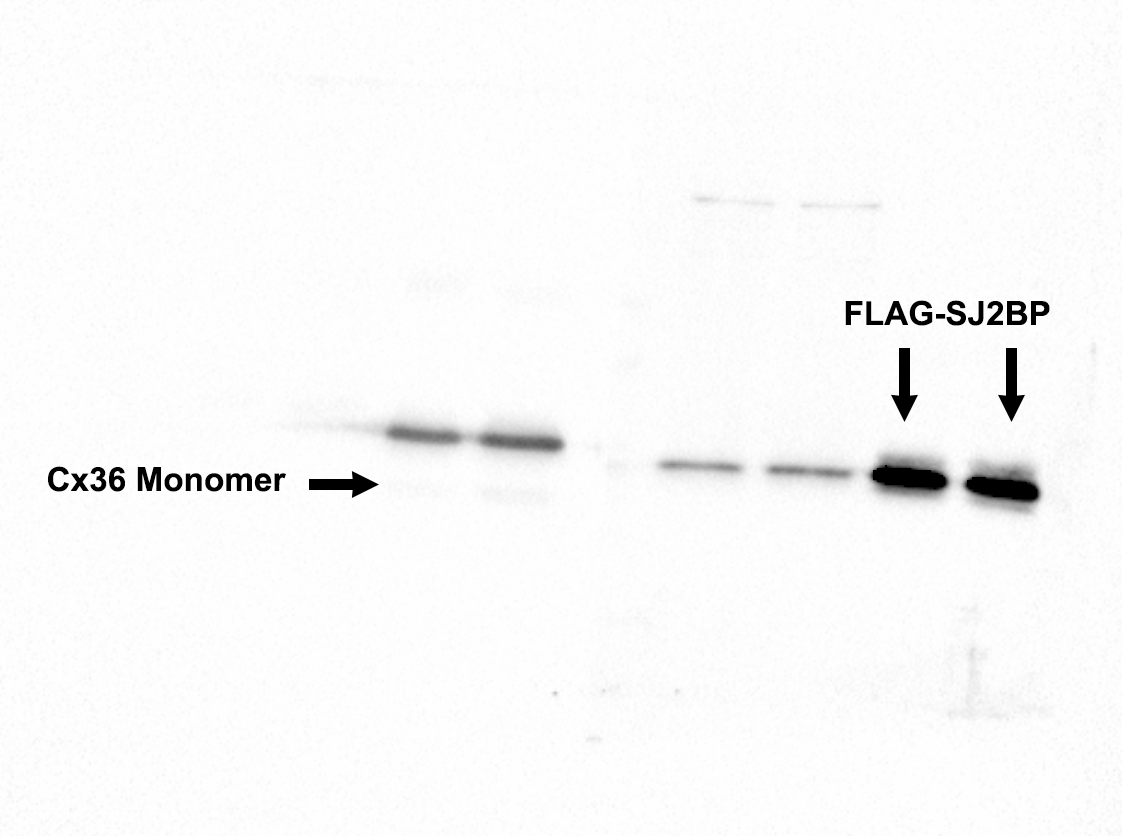

Supplement: Figure 5—source data 1. — Relevant bands were labeled with arrows. [file elife-105935-fig5-data1.zip › labelled blots/Figure 5B_Cx36 and SJ2BP II_labelled.tif]

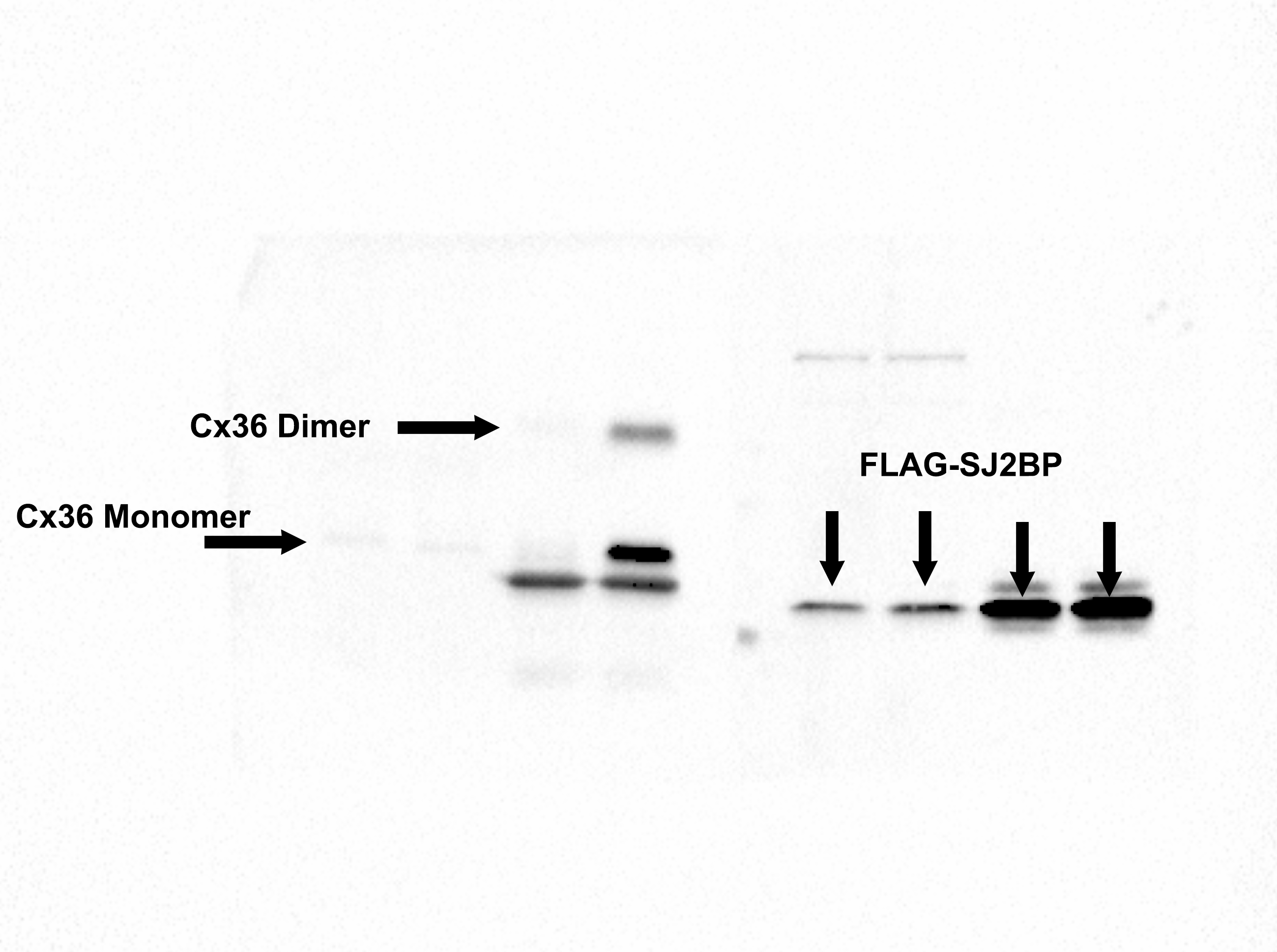

Supplement: Figure 5—source data 1. — Relevant bands were labeled with arrows. [file elife-105935-fig5-data1.zip › labelled blots/Figure 5B_Cx36 and SJ2BP_labelled II.tif]

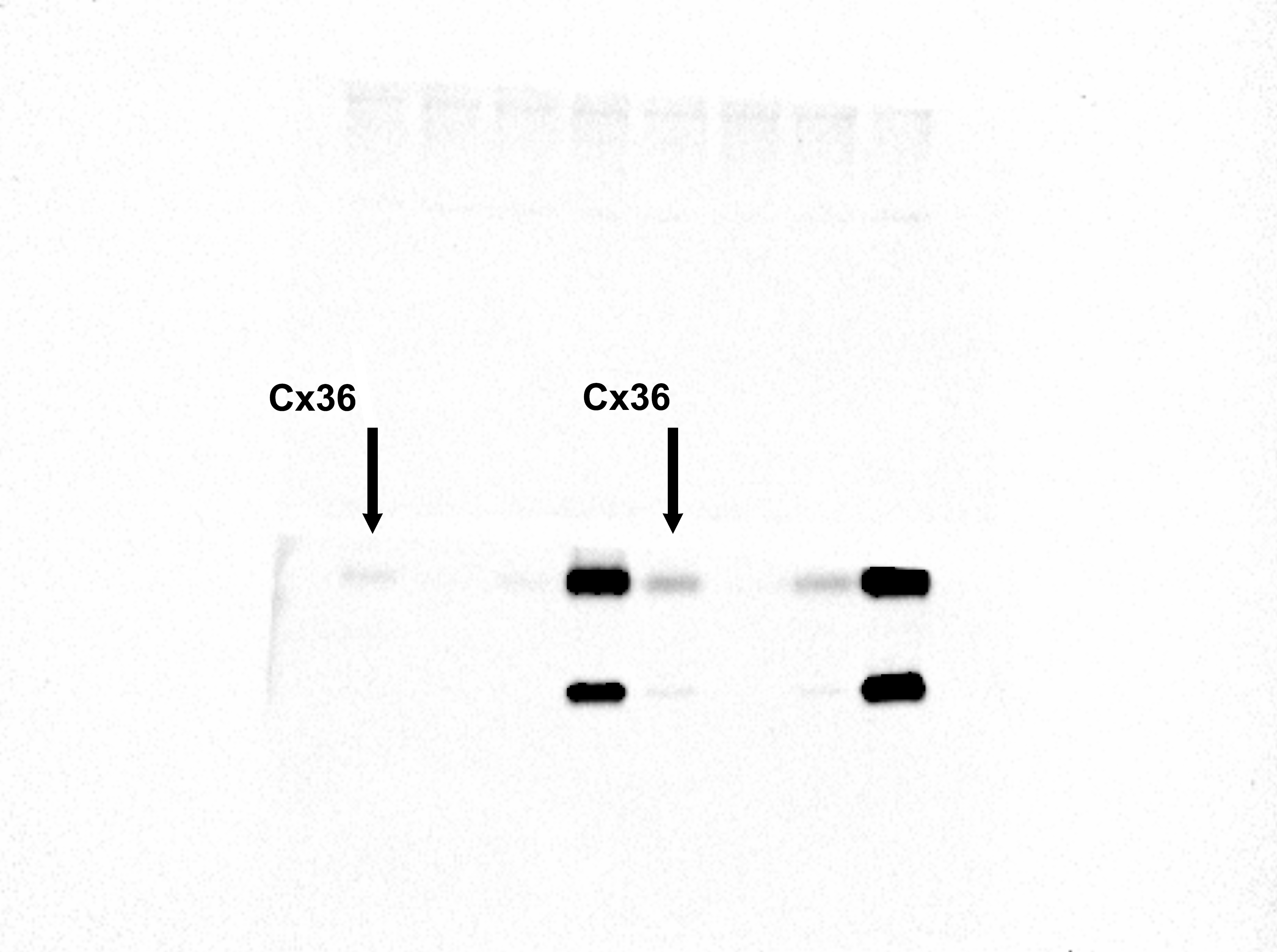

Supplement: Figure 5—source data 1. — Relevant bands were labeled with arrows. [file elife-105935-fig5-data1.zip › labelled blots/Figure 5b_Cx36_labelled.tif]

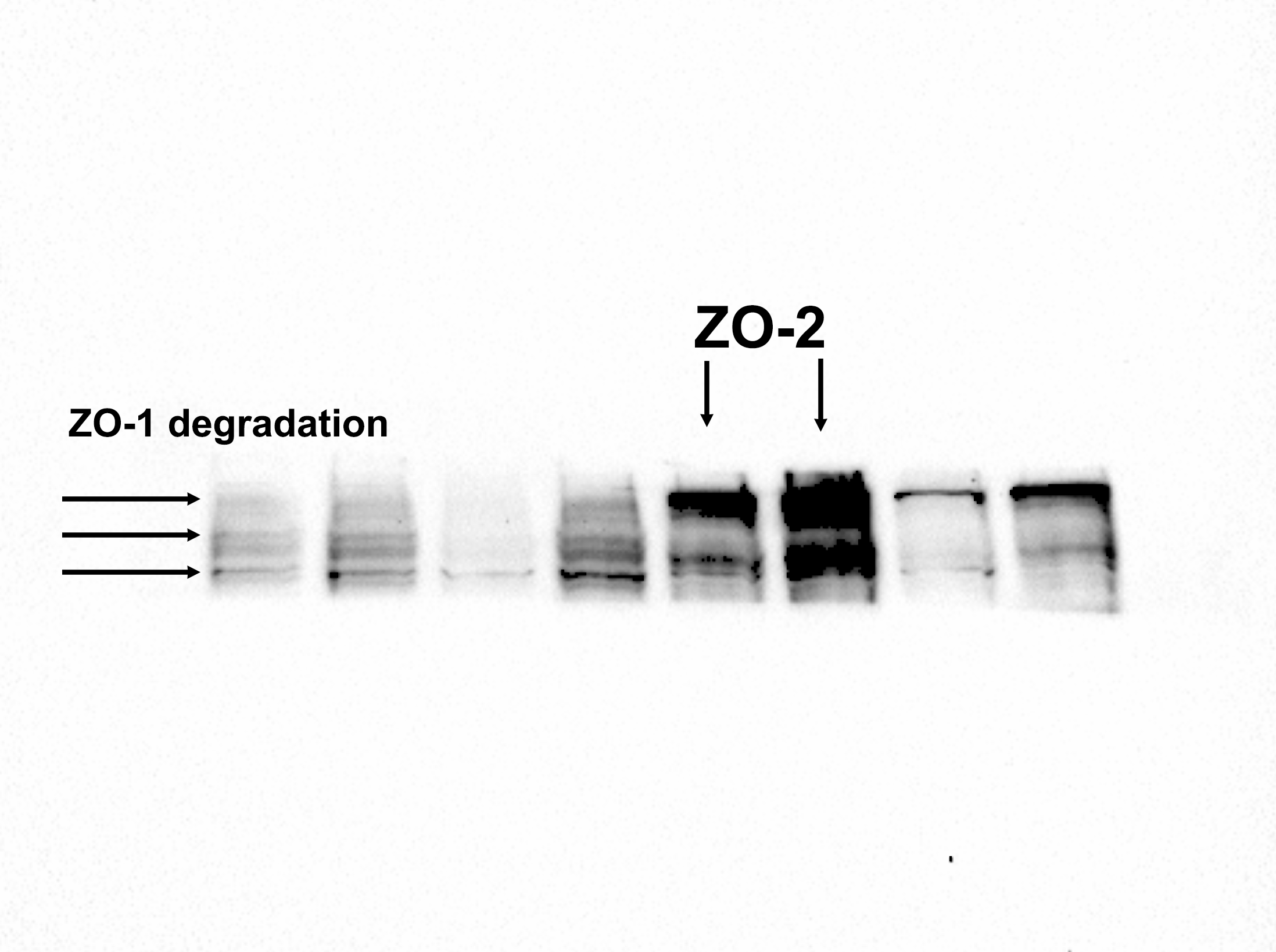

Supplement: Figure 5—source data 1. — Relevant bands were labeled with arrows. [file elife-105935-fig5-data1.zip › labelled blots/Figure 5B_GFP_Input_labelled.tif]

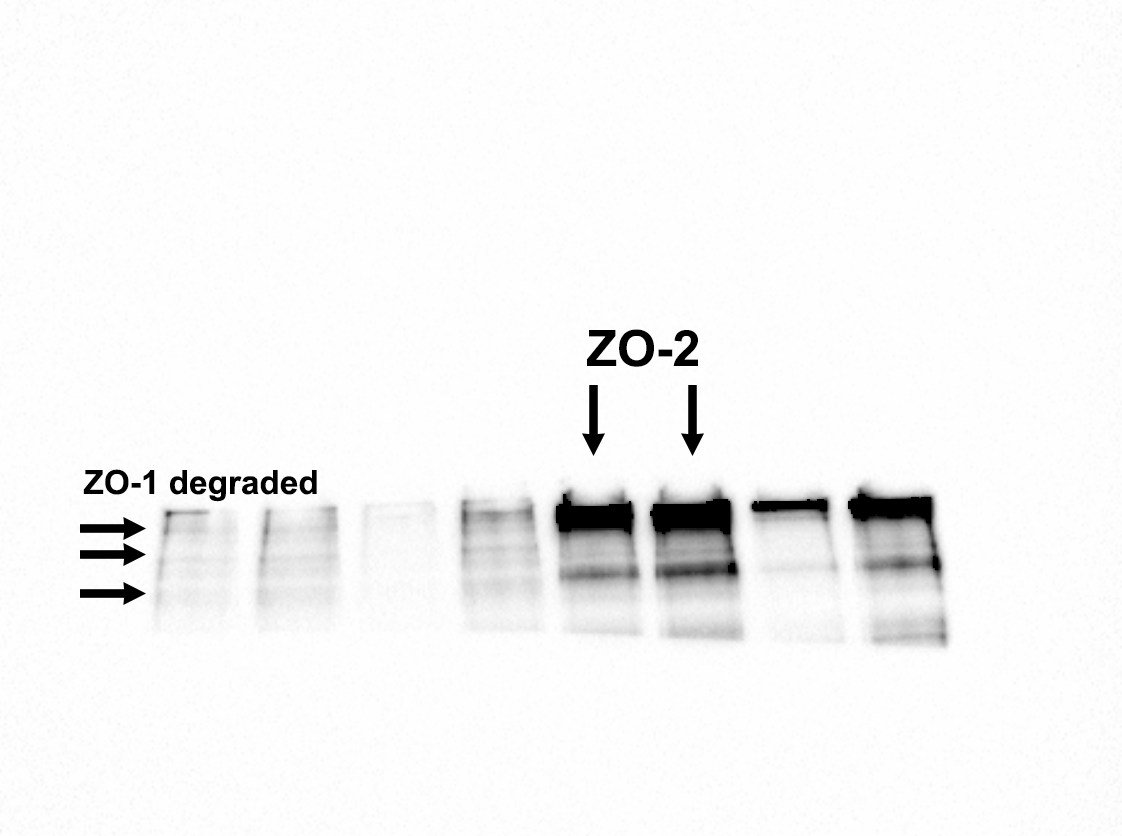

Supplement: Figure 5—source data 1. — Relevant bands were labeled with arrows. [file elife-105935-fig5-data1.zip › labelled blots/Figure 5B_GFP_labelled_IP.tif]

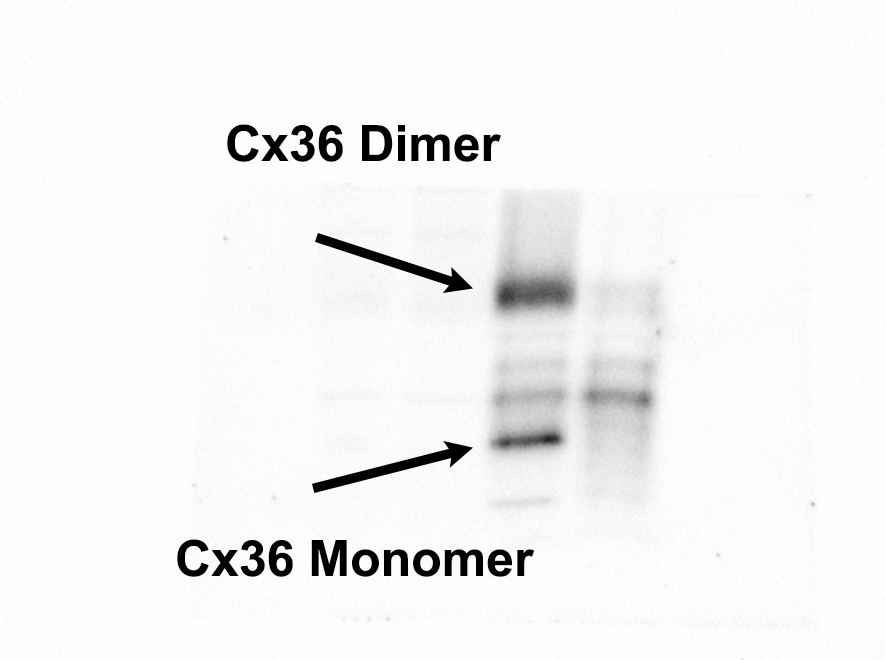

Supplement: Figure 5—source data 1. — Relevant bands were labeled with arrows. [file elife-105935-fig5-data1.zip › labelled blots/Figure 5B_Sipa and Cx36_labelled.tif]

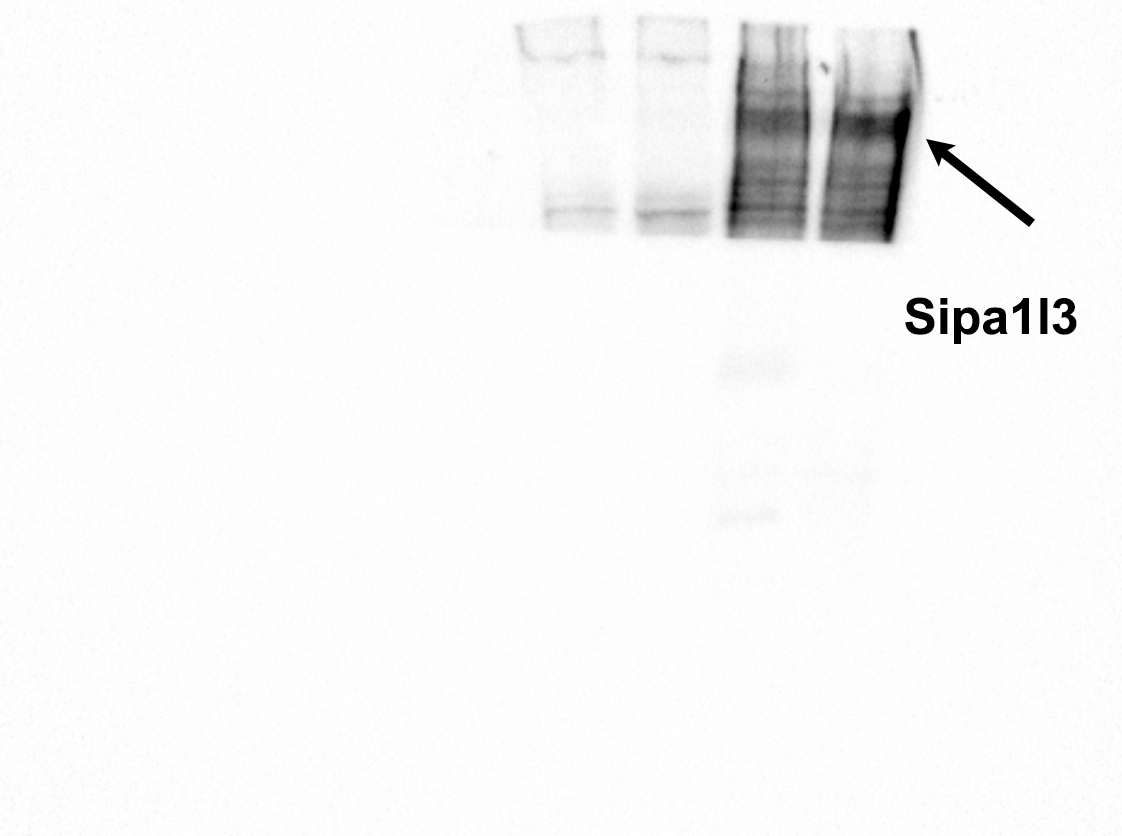

Supplement: Figure 5—source data 1. — Relevant bands were labeled with arrows. [file elife-105935-fig5-data1.zip › labelled blots/Figure 5B_SIPA1l3 labelled.tif]

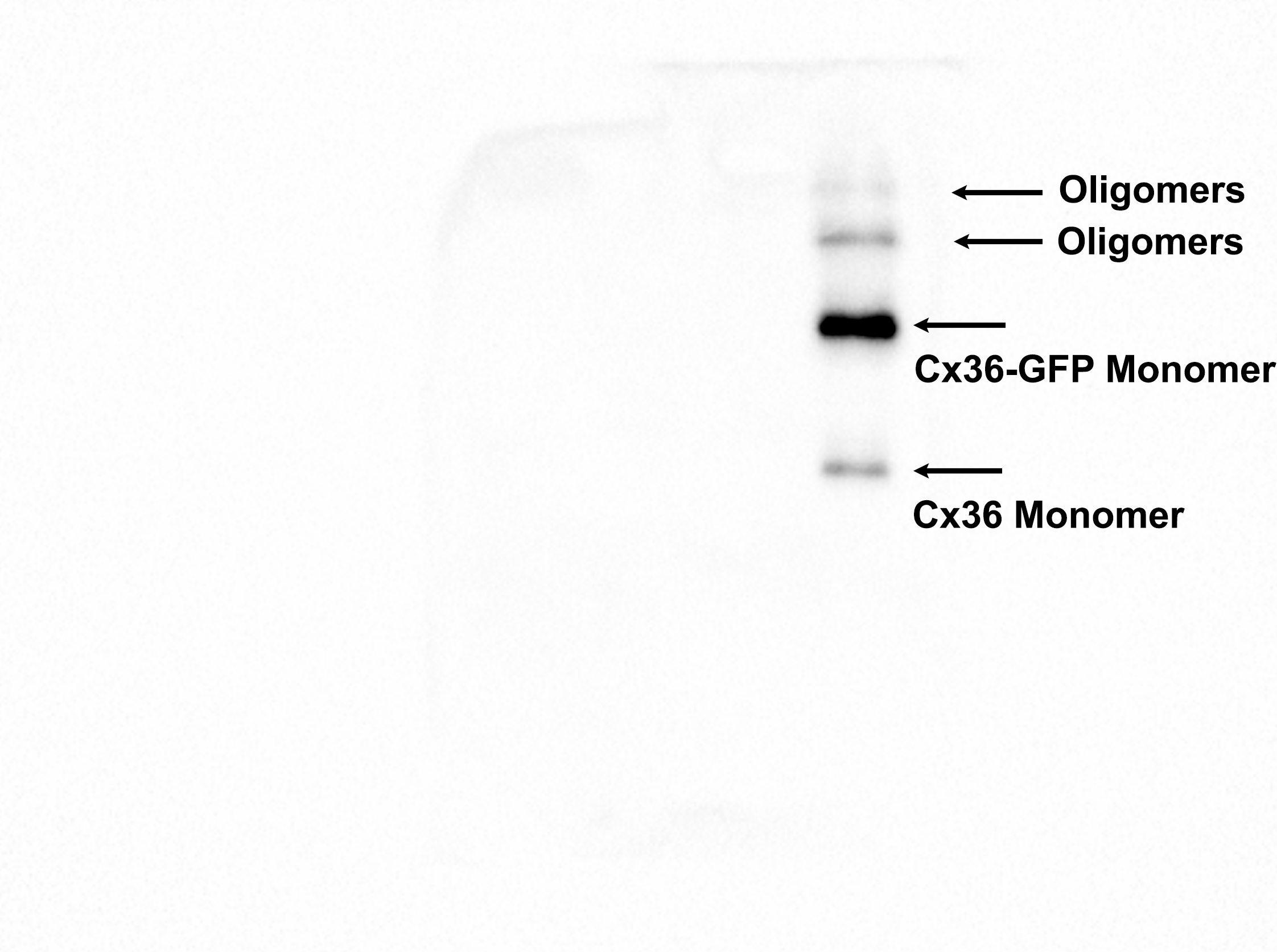

Supplement: Figure 5—source data 1. — Relevant bands were labeled with arrows. [file elife-105935-fig5-data1.zip › labelled blots/Figure 5C_Cx36_labelled.tif]

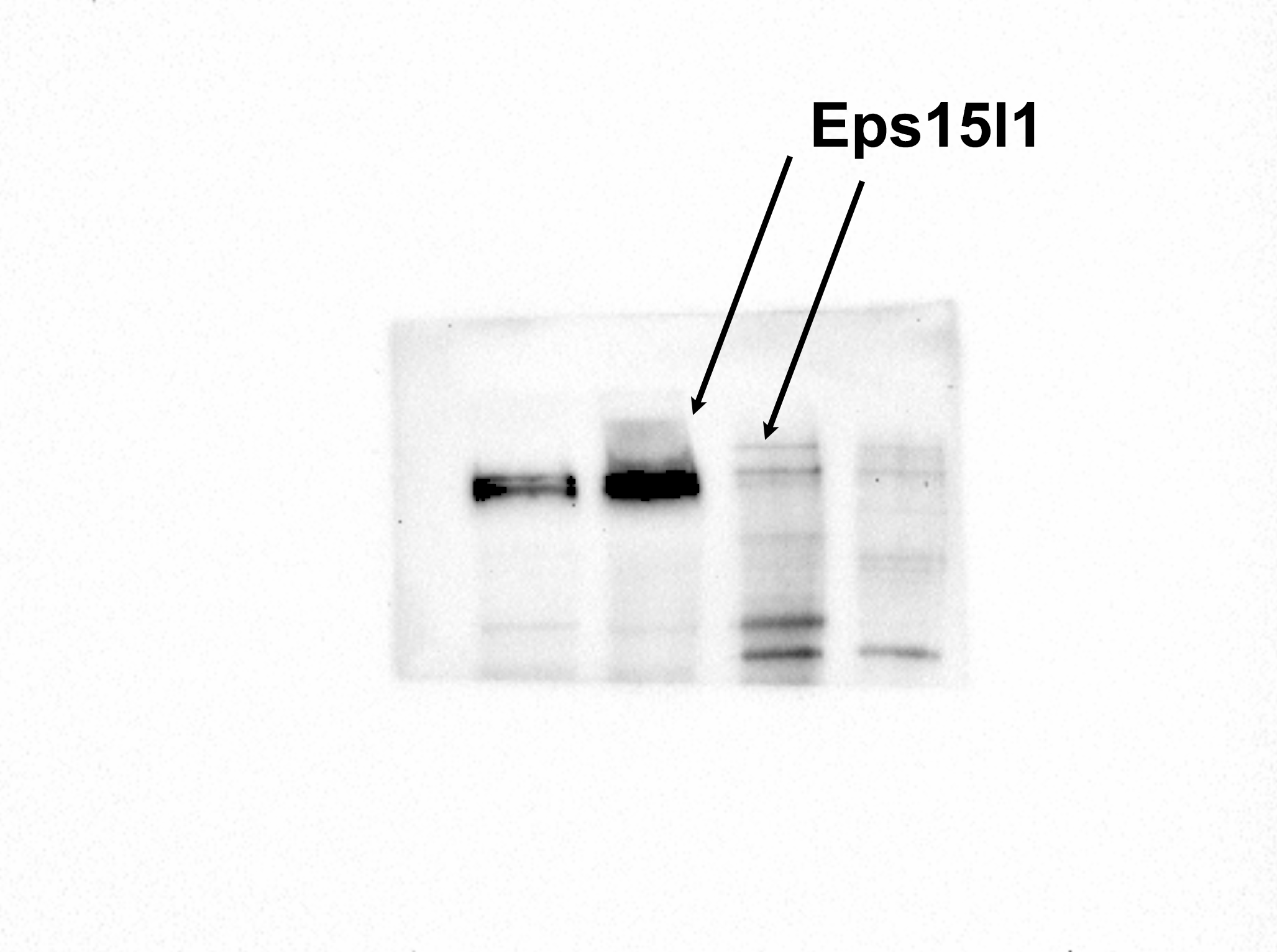

Supplement: Figure 5—source data 1. — Relevant bands were labeled with arrows. [file elife-105935-fig5-data1.zip › labelled blots/Figure 5C_EPS15l1_labelled.tif]

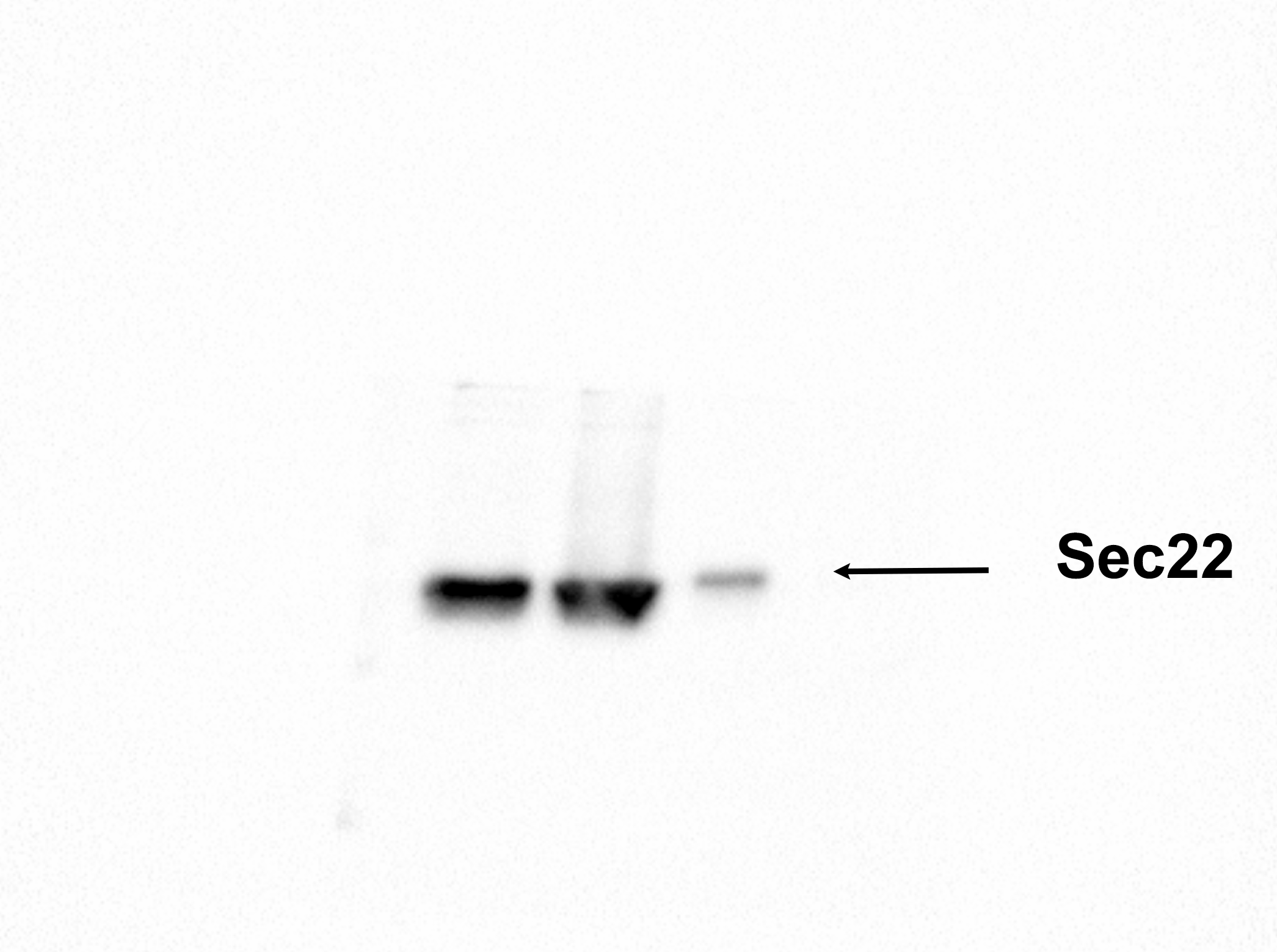

Supplement: Figure 5—source data 1. — Relevant bands were labeled with arrows. [file elife-105935-fig5-data1.zip › labelled blots/Figure 5C_Sec22_labelled.tif]

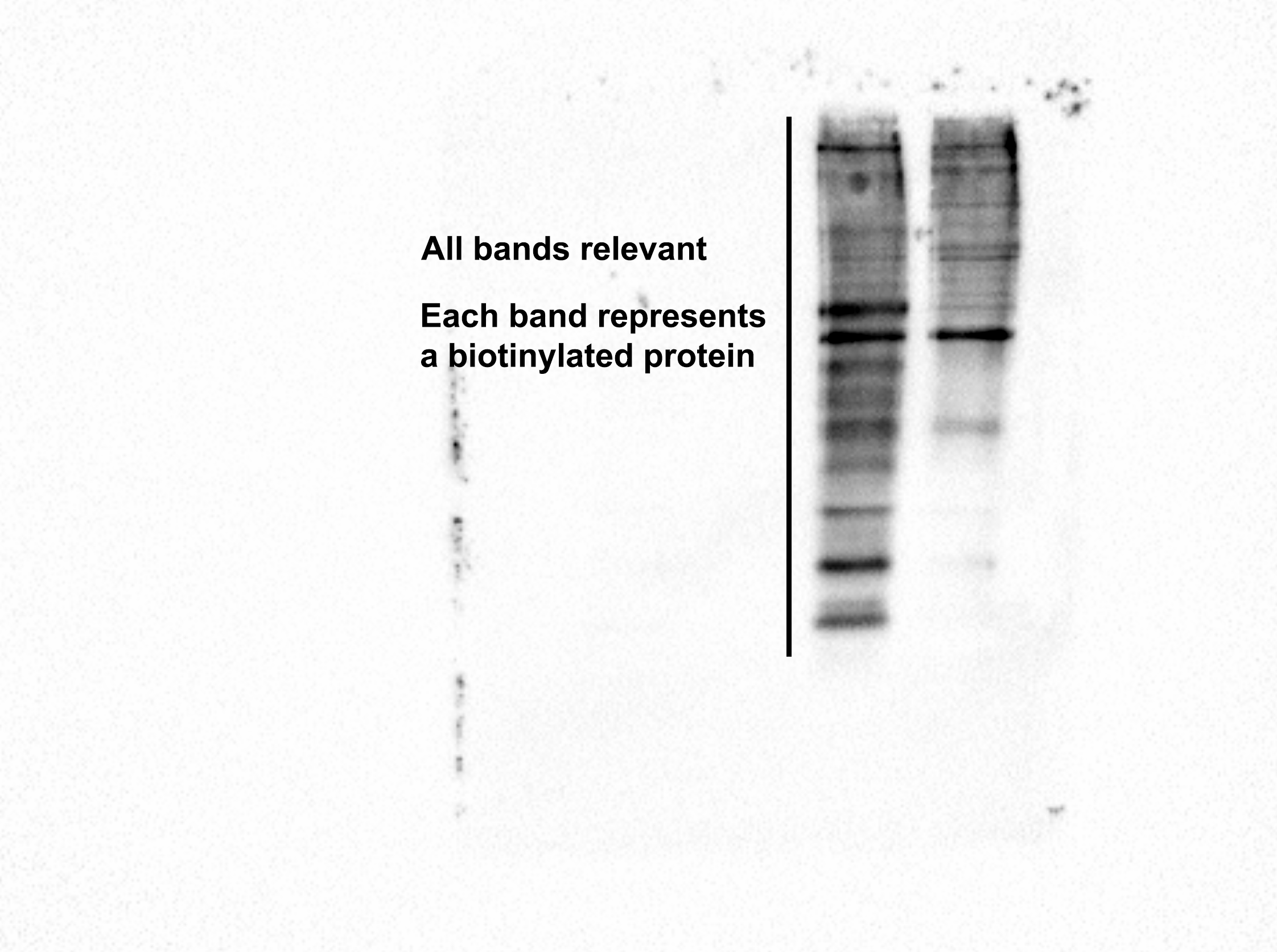

Supplement: Figure 5—source data 1. — Relevant bands were labeled with arrows. [file elife-105935-fig5-data1.zip › labelled blots/Figure 5C_Streptavidin_labelled.tif]

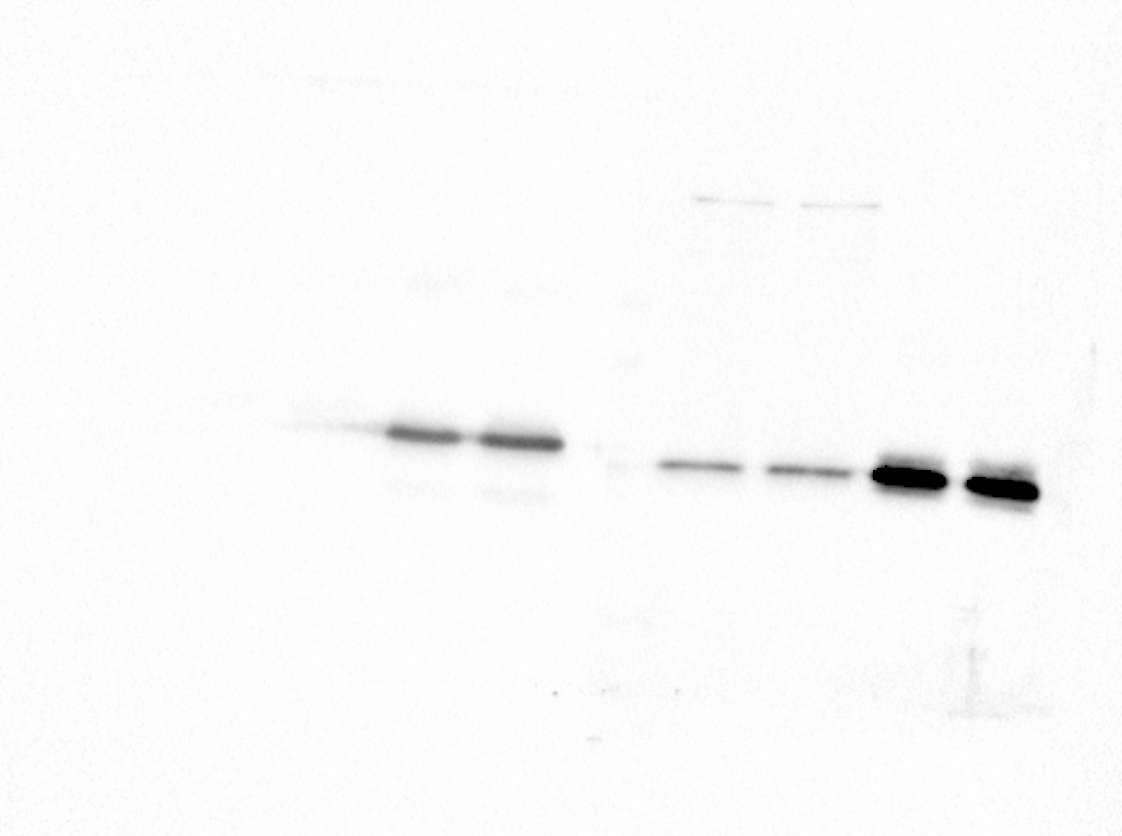

Supplement: Figure 5—source data 2. [file elife-105935-fig5-data2.zip › Unlabelled blots/Figure 5B_Cx36 and SJ2BP I.tif]

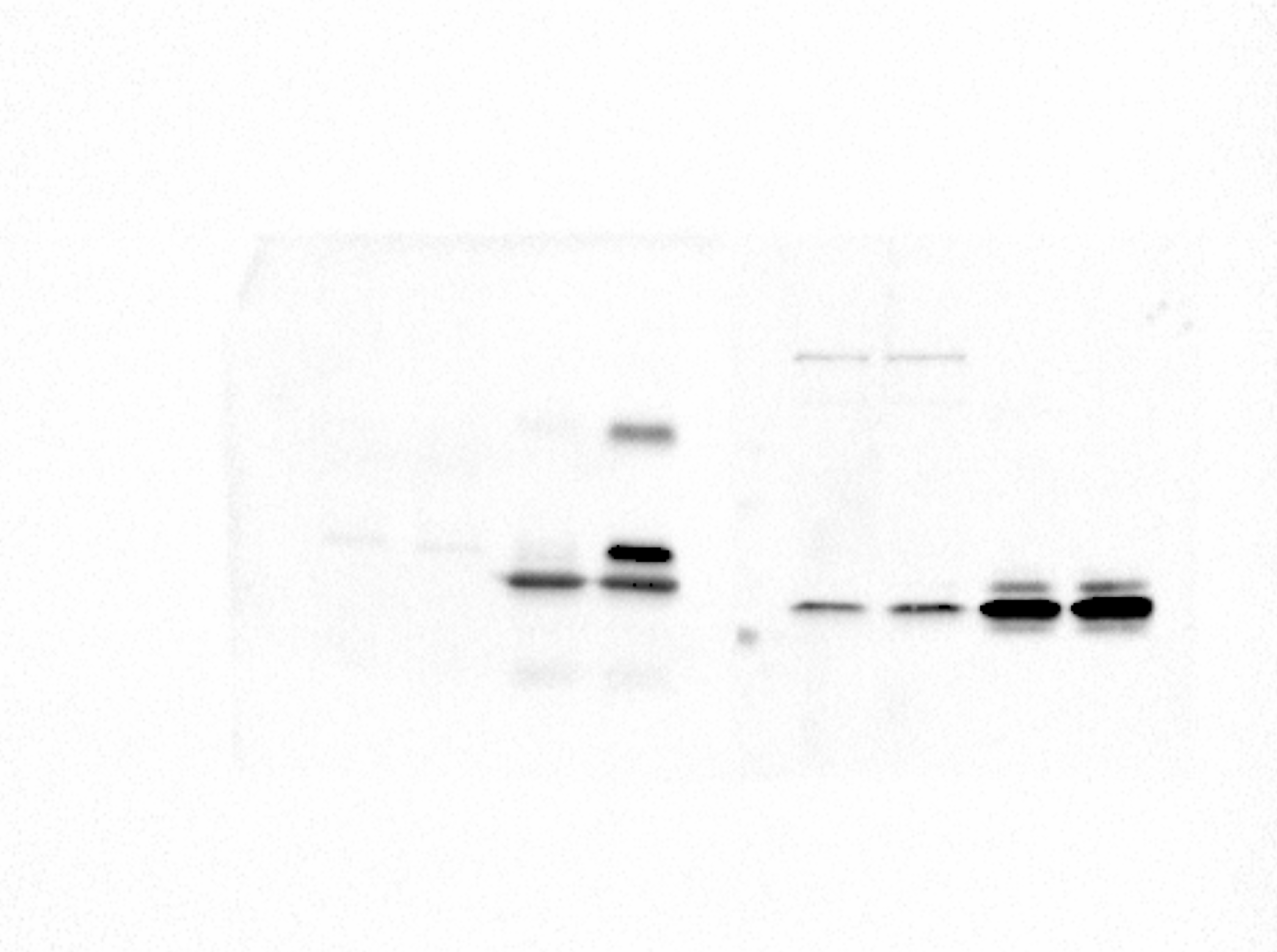

Supplement: Figure 5—source data 2. [file elife-105935-fig5-data2.zip › Unlabelled blots/Figure 5B_Cx36 and SJ2BP II.tif]

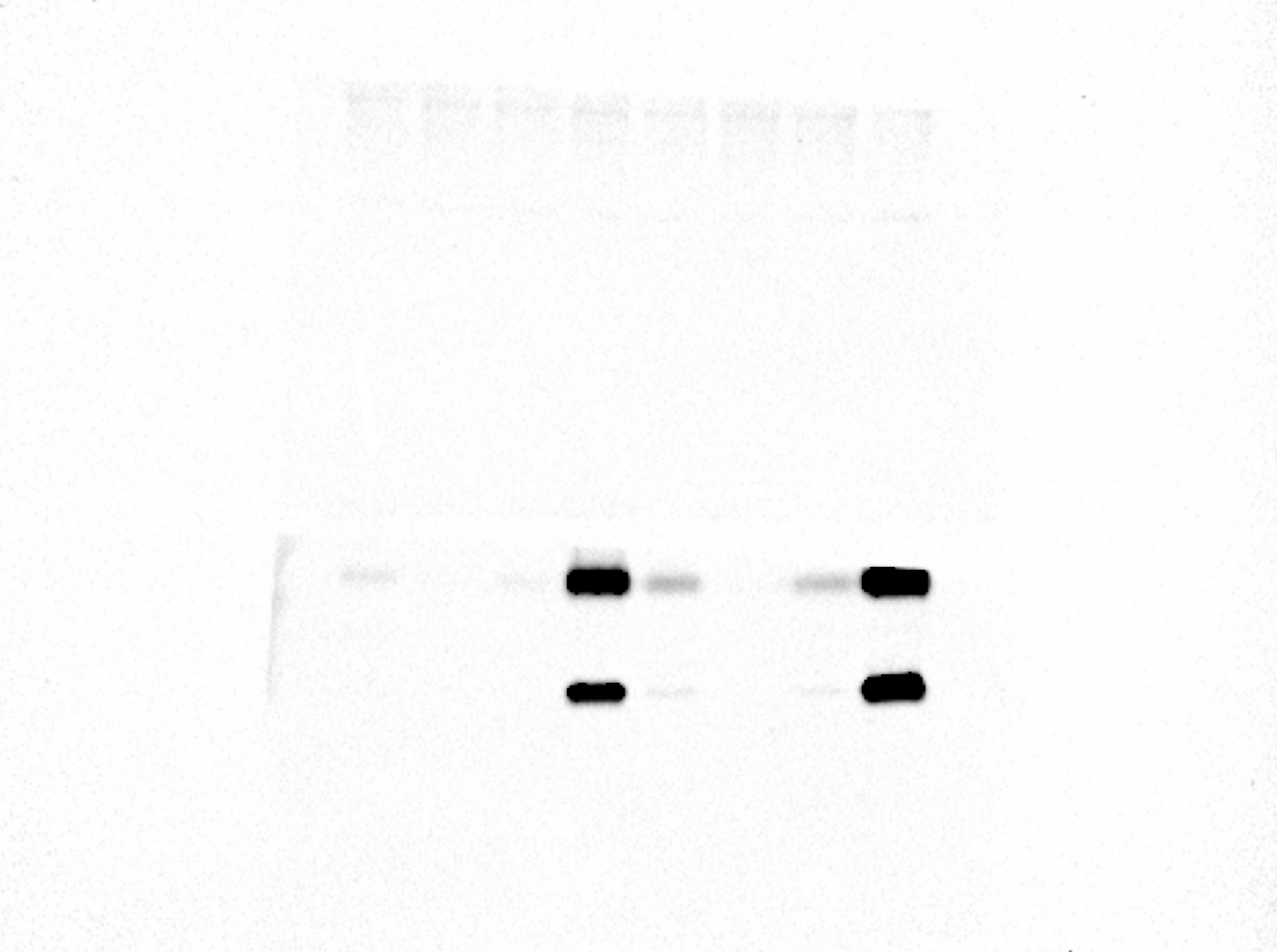

Supplement: Figure 5—source data 2. [file elife-105935-fig5-data2.zip › Unlabelled blots/Figure 5b_Cx36.tif]

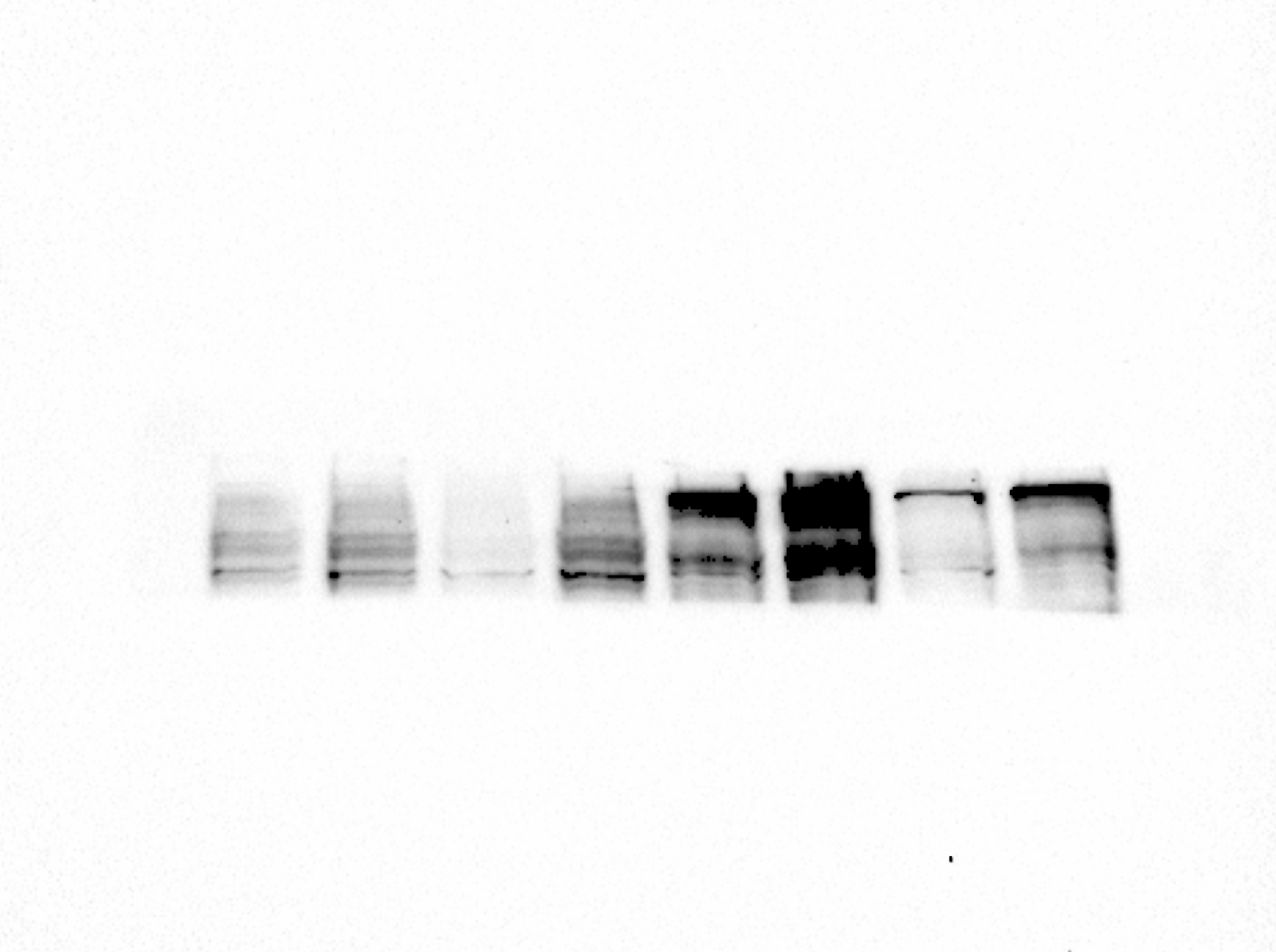

Supplement: Figure 5—source data 2. [file elife-105935-fig5-data2.zip › Unlabelled blots/Figure 5B_GFP_Input.tif]

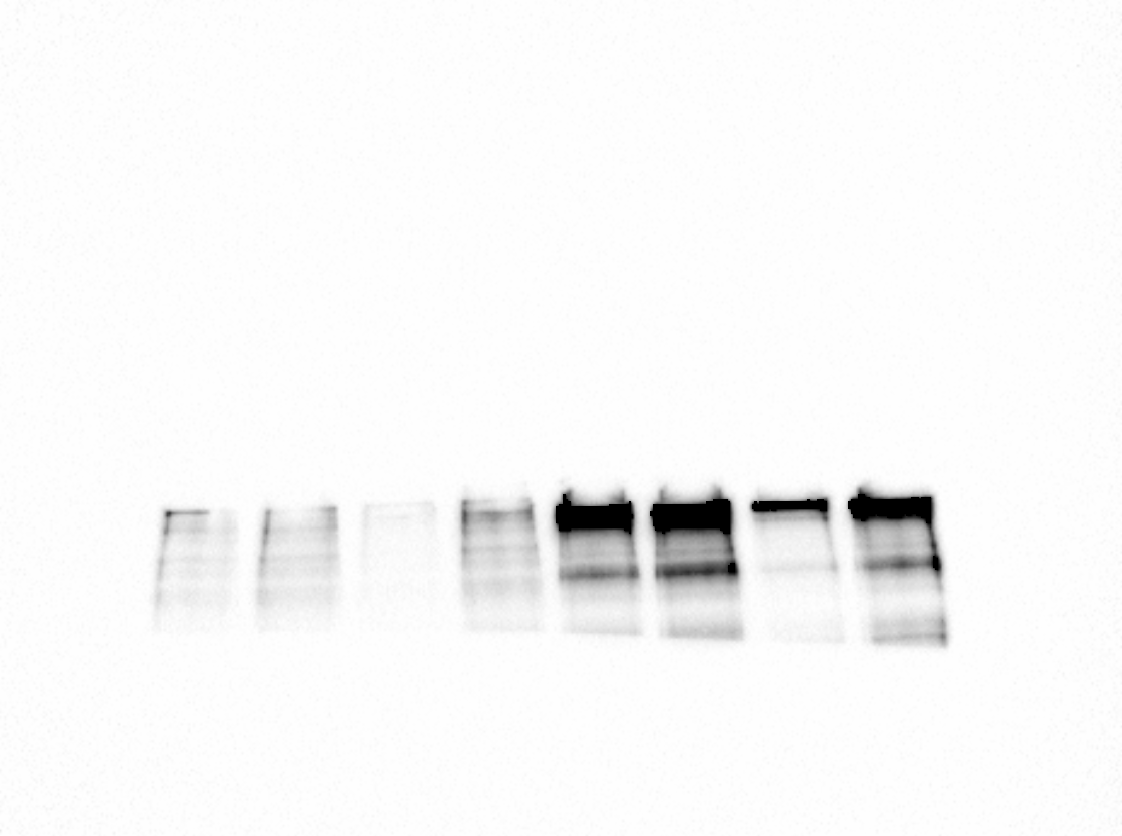

Supplement: Figure 5—source data 2. [file elife-105935-fig5-data2.zip › Unlabelled blots/Figure 5B_GFP_IP.tif]

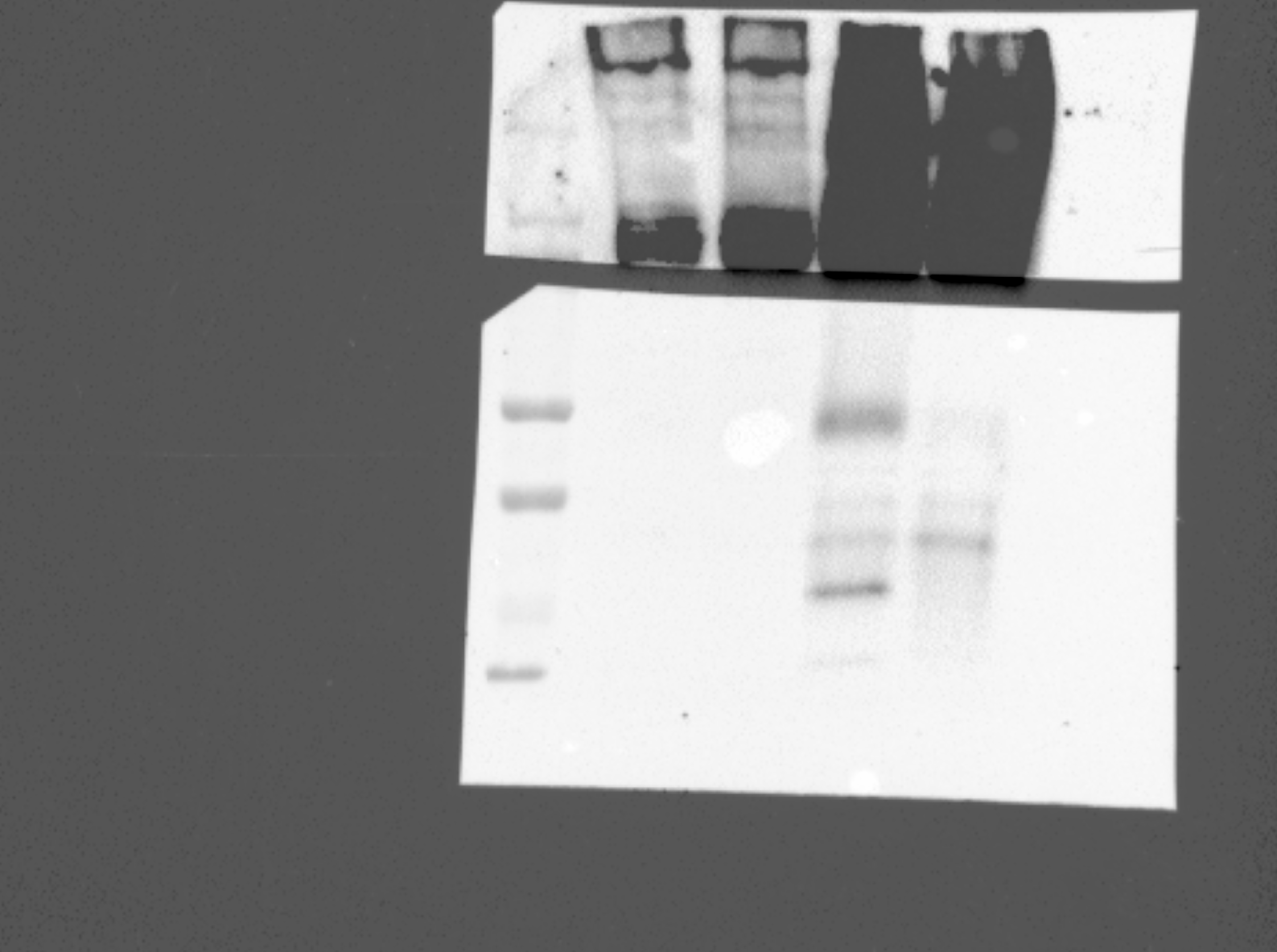

Supplement: Figure 5—source data 2. [file elife-105935-fig5-data2.zip › Unlabelled blots/Figure 5B_Sipa1l3 and Cx36 with marker.tif]

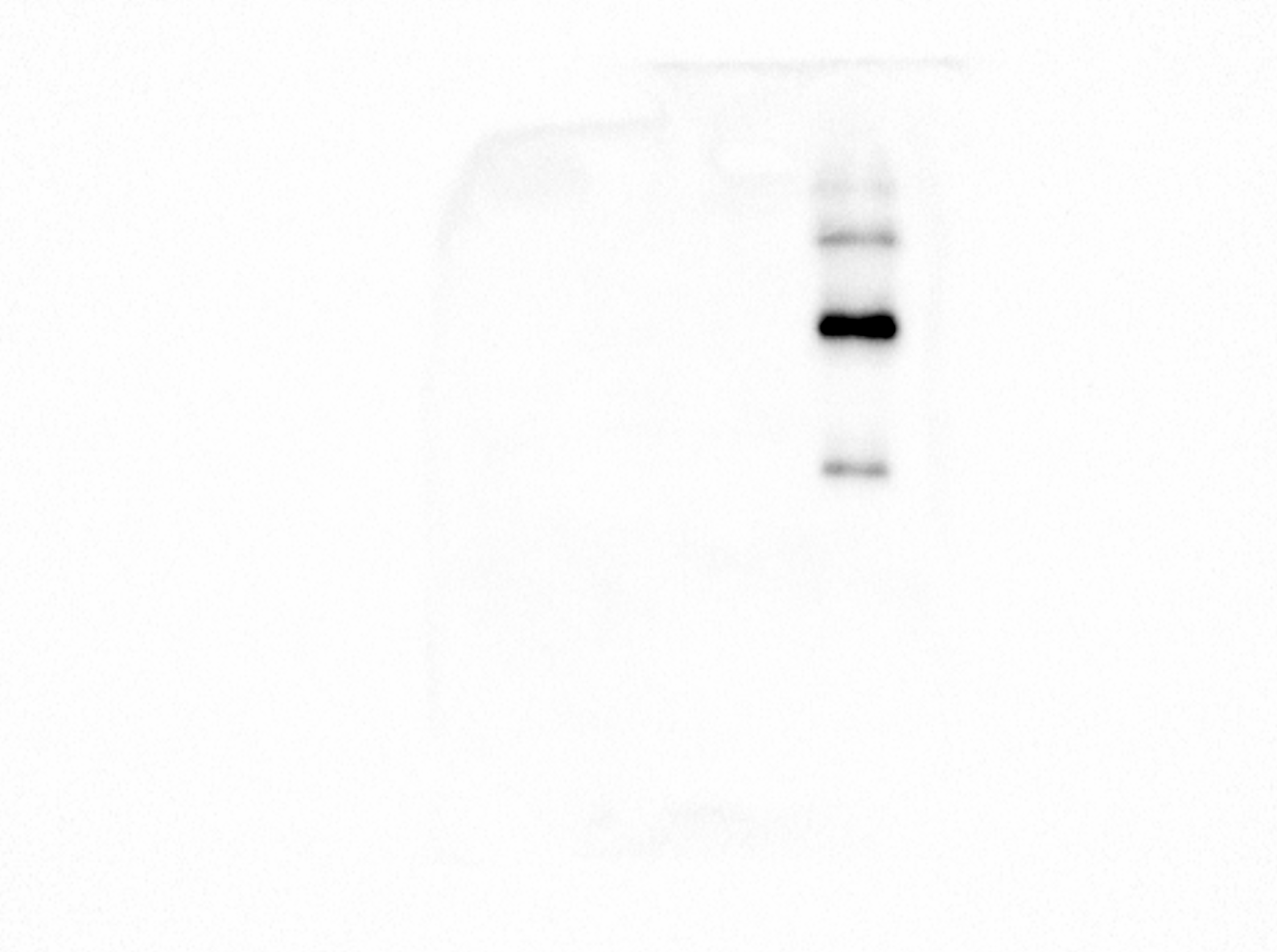

Supplement: Figure 5—source data 2. [file elife-105935-fig5-data2.zip › Unlabelled blots/Figure 5C_Cx36.tif]

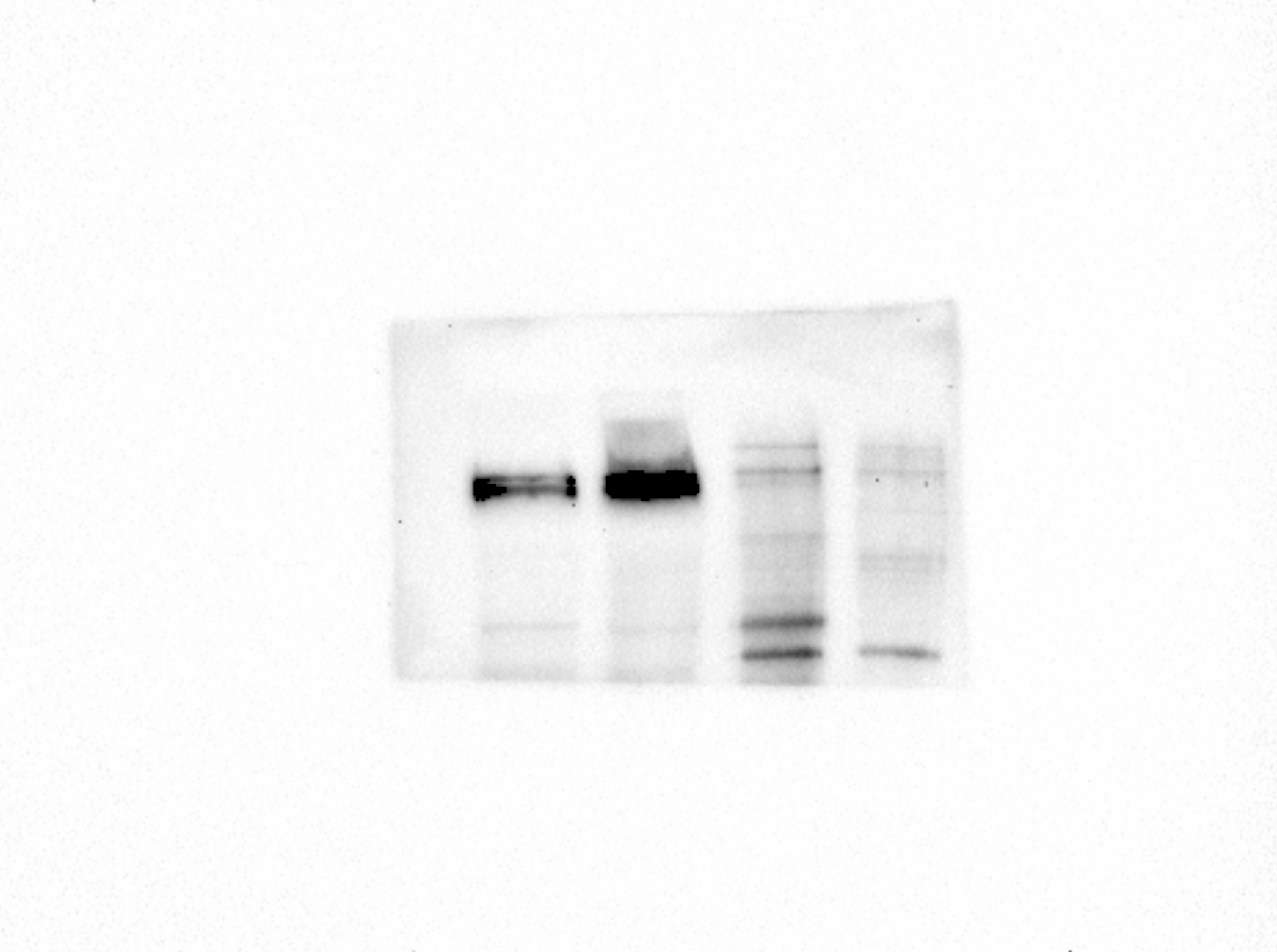

Supplement: Figure 5—source data 2. [file elife-105935-fig5-data2.zip › Unlabelled blots/Figure 5C_EPS15l1.tif]

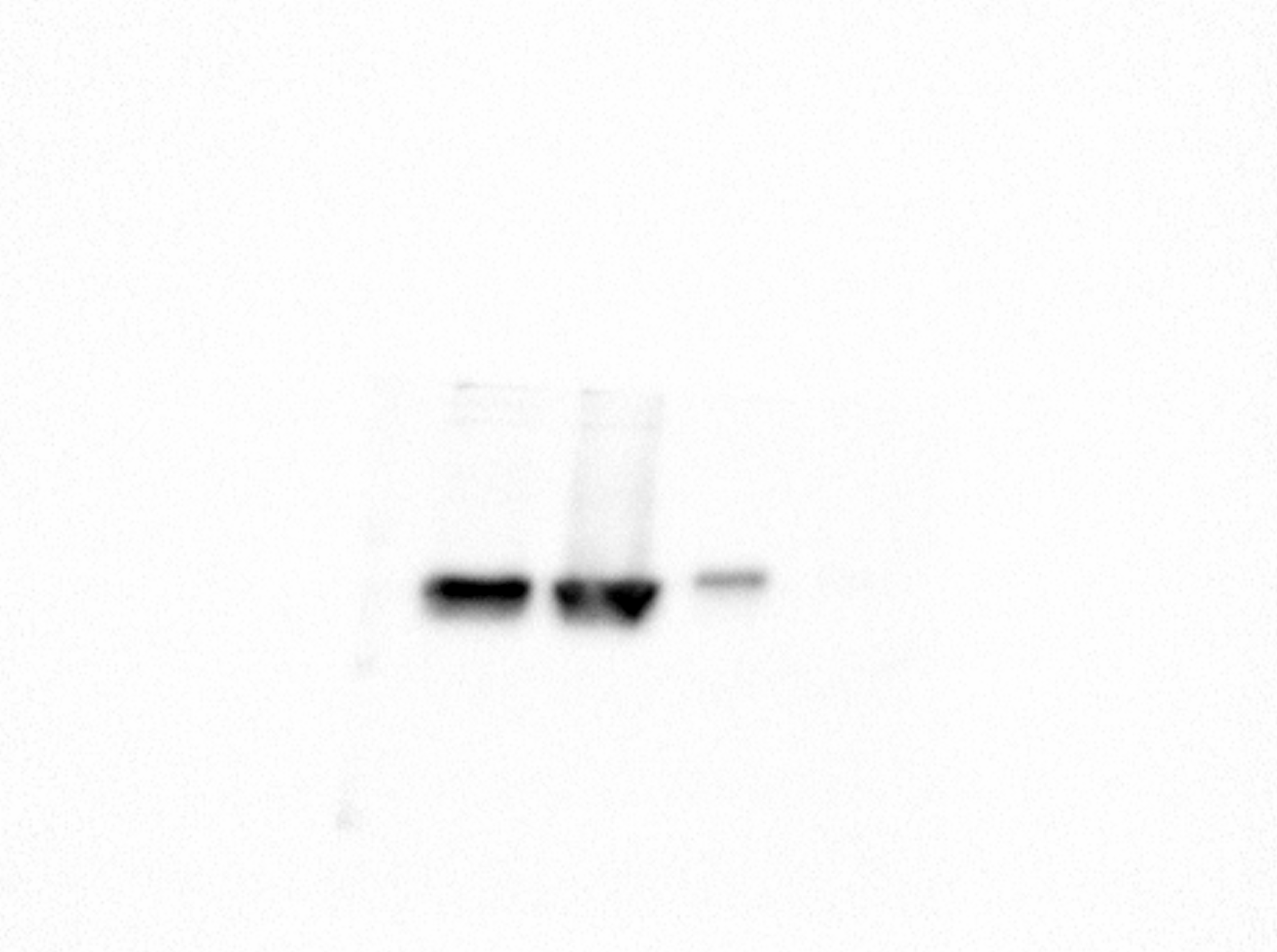

Supplement: Figure 5—source data 2. [file elife-105935-fig5-data2.zip › Unlabelled blots/Figure 5C_Sec22.tif]

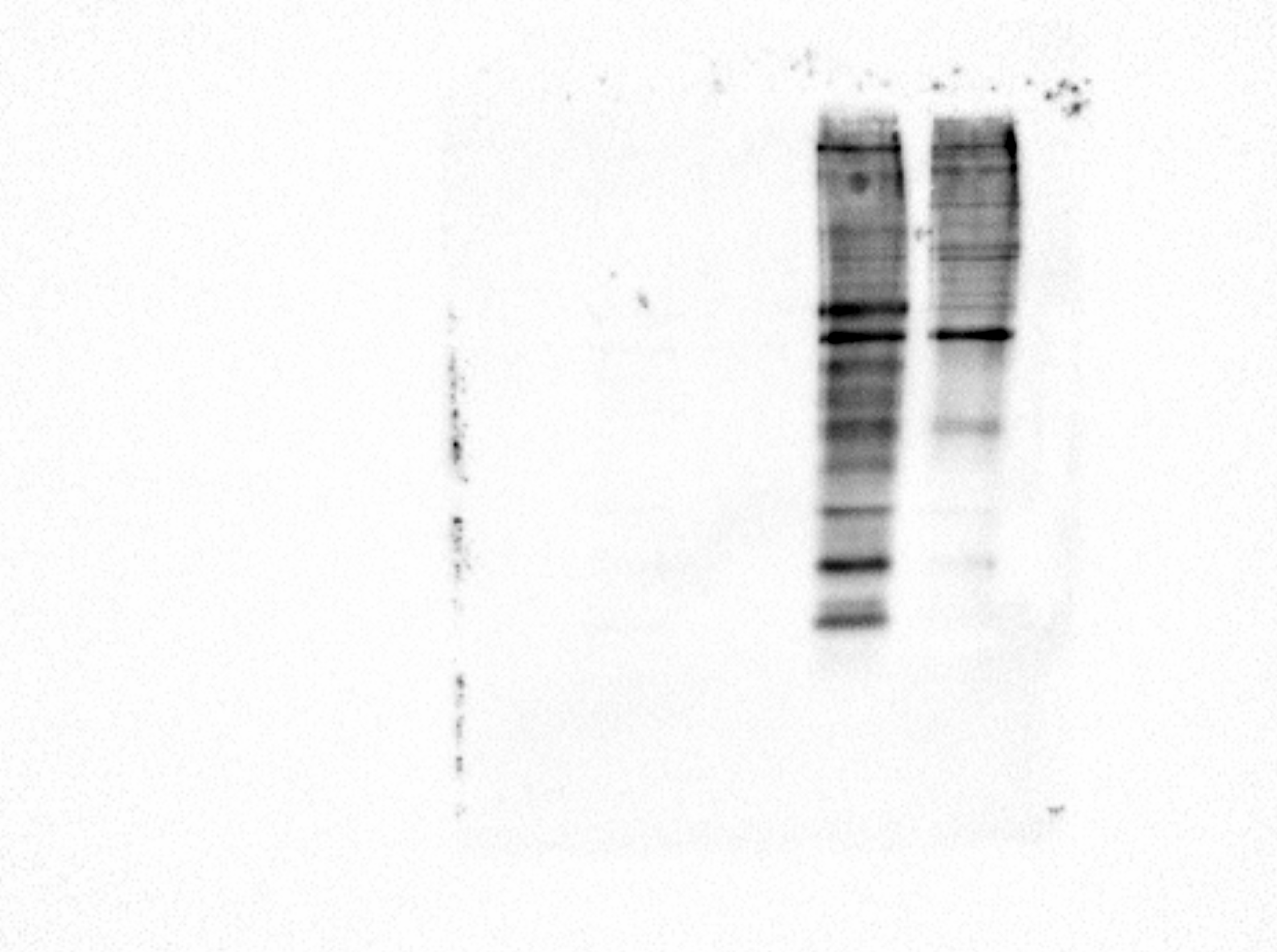

Supplement: Figure 5—source data 2. [file elife-105935-fig5-data2.zip › Unlabelled blots/Figure 5C_Streptavidin.tif]
