# Supplementary material for: Uncovering the electrical synapse proteome in retinal neurons via in vivo proximity labeling
Source: eLife. 2026 May 20;14:RP105935. doi: 10.7554/eLife.105935 (PMC13189625; doi:10.7554/eLife.105935)
Supplement: Supplementary file 3. [file elife-105935-supp3.docx]

Supplementary Table 3 – Conservation of electrical synapse proteome

| **Protein** | **Zebrafish Cx35b (predominantly photoreceptor)** | **Mouse Cx36 (predominantly AII amacrine cell)** |
| --- | --- | --- |
| **Scaffolds** |  |  |
| Tjp1a (ZO1a) | +++ | +++ |
| Tjp1b | +++ |  |
| Tjp2a (ZO2a) | +++ | ++ |
| LOC103911473 (tjp2-like) | ++ |  |
| Sipa1l3 | - | +++ |
| Sipa1l1 | + | + |
| Mpdz (Mupp1) | ++ | - |
| Shank2b | + | ++ |
| Dlg4 (Psd95) | + | * |
| Mllt4a (Afadin) | ++ | + |
| Cingulin | - | + |
| LOC556872 (Magi1) | ++ | - |
| Magixb | ++ | - |
| Magixa | + |  |
|  |  |  |
| **Gap junction** |  |  |
| Gjd2 (Cx36) | - | +++ |
| LOC100331216 (gjd1b) | ++ | - |
|  |  |  |
| **Cell-cell junction** |  |  |
| Pkp4 | + | ++ |
| Ncam1 |  | +++ |
| Adgrb1 | - | + |
|  |  |  |
| **Endocytosis** |  |  |
| Eps15l1 | ++ | ++ |
| Snap91 (AP180) | + | ++ |
| Myo6a | ++ | ++ |
| Myo6b | ++ |  |
| Hip1r | - | + |
| Picalmb | + | ++ |
| Synrg | ++ | ++ |
| Itsn | - | ++ |
| Epn1 | - | ++ |
|  |  |  |
| **Trafficking** |  |  |
| Stxbp1 | - | ++ |
| Stxbp4 | +++ | - |
| Sec22bb | + | + |
| Golga4 | - | ++ |
|  |  |  |
| **Cytoskeleton regulators** |  |  |
| Syngap1b | + | + |
| Arhgap32 | - | ++ |
| Map6 | - | ++ |
| Dock7 | - | ++ |
| Gprin1 | - | + |
|  |  |  |
| **Protein kinase signaling** |  |  |
| Camk2d | * | ++ |
| Camk2g | - | ++ |
| Camk2b | - | ++ |
| Aak1 | - | + |
| Tnikb | ++ | - |
| Akap8l | + | * |
|  |  |  |
| **Cell polarity/apical junction** |  |  |
| si:ch211-13f8.1 (KIAA1614 orthologue) | ++ | - |
| si:dkey-121a11.3 (KIAA1614) | ++ |  |
| Ajm1 | - | + |

+++ Mean area > 1x10^8^

++ Mean area > 1x10^7^

+ Mean area > 1x10^6^

- Not detected

* Present but below threshold
